# Supplementary material for: A Basal Forebrain-Cingulate Circuit in Macaques Decides It Is Time to Act
Source: Neuron. 2020 Jan 22;105(2):370–384.e8. doi: 10.1016/j.neuron.2019.10.030 (PMC6975166; doi:10.1016/j.neuron.2019.10.030)
Supplement: Document S1. Figures S1–S7 and Table S1 [file mmc1.pdf]

Neuron, Volume *105*

Supplemental Information

A Basal Forebrain-Cingulate Circuit

in Macaques Decides It Is Time to Act

Nima Khalighinejad, Alessandro Bongioanni, Lennart Verhagen, Davide Folloni, David Attali, Jean-Francois Aubry, Jerome Sallet, and Matthew F.S. Rushworth

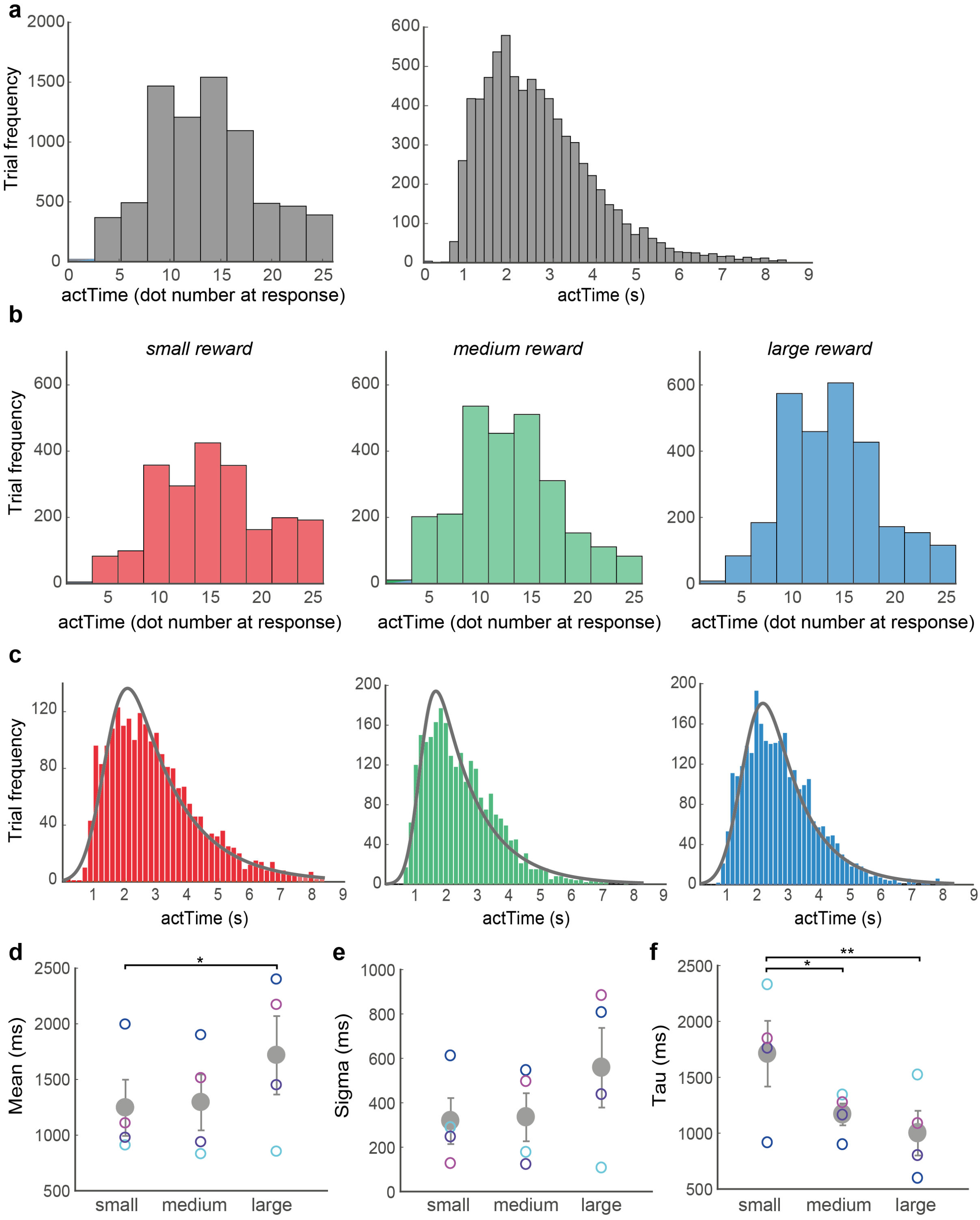
a
Trial frequency
actTime (dot number at response)
actTime (s)
b
small reward
medium reward
large reward
Trial frequency
actTime (dot number at response)
c
Trial frequency
actTime (s)
d
Mean (ms)
small
medium
large
e
Sigma (ms)
f
Tau (ms)

Figure S1. Distribution of *actTime*. Related to Figure 2. (a) Distribution of *actTime* across all trials. The histogram shows a wide range of *actTime* in dot number (left panel) and seconds (right panel); while on some trials animals were impulsive and responded after emergence of the first 2-3 dots, on many trials they waited till the very last dot before responding. (b) To get a better understanding of the U-shaped relationship between reward magnitude and *actTime* we looked at the distribution of *actTime* for different levels of reward**.** The histograms suggest that despite the quadratic effect of reward magnitude on *actTime*, the distribution of *actTime* has a distinct pattern in small and large reward trials: *actTimes* are distributed more uniformly in the former compared to the latter. (c) To capture the extended tail of the time-to-act distribution we fitted an exponentially modified Gaussian distribution on each animal's time-to-act in milliseconds, separately for small, medium and large reward magnitude trials. The ex-Gaussian model is derived via convolution of an exponential distribution and a normal distribution and captures response time distributions well (Lewandowsky and Farrell, 2011). It assumes that response time can be divided into two independent components: 1) the time taken to make a decision, assumed to be exponentially distributed (Andrews and Heathcote, 2001; Balota et al., 2008), and 2) processes supplementary to the decision assumed to be Gaussian (Luce, 1991). (d-f) The model has three free parameters: mean and standard deviation (sigma) of the Gaussian distribution and tau, the rate of drop-off of the exponential distribution. We found a significant main effect of reward magnitude on the mean ($X^2(2)=7.51$, $P=0.023$) (d), but not on the standard deviations of the Gaussian components ($X^2(2)=3.68$, $P=0.16$) (e). However, tau was significantly higher in small reward compared to large reward magnitude trials (main effect, $X^2(2)=12.48$, $P=0.002$; small vs. large, $\beta=711\pm156$, $t(6)=4.55$, $P=0.004$; small vs. medium, $\beta=543\pm156$, $t(6)=3.47$, $P=0.013$) (f). This suggests animals made more deliberate decisions when offered large compared to small rewards; decisions about when to act were relatively tightly clustered around long *actTimes*. However, when confronted with small rewards, animals appeared relatively indifferent and there was considerable variation in *actTime*, sometimes waiting till the very last moment and responding just before the trial

ended. Error bars represent standard error of the mean, and the grey circles illustrate the group mean. The model was separately fitted on pooled data (time-to-act in milliseconds) from each animal. Fitting was performed in MATLAB (MathWorks, MA, USA) by using maximum likelihood estimation and a bounded Simplex algorithm. Each colour ring represents the best fit for each animal. Multilevel ANOVA followed by pairwise t-test. * $P<0.05$, ** $P<0.01$, *** $P<0.001$.

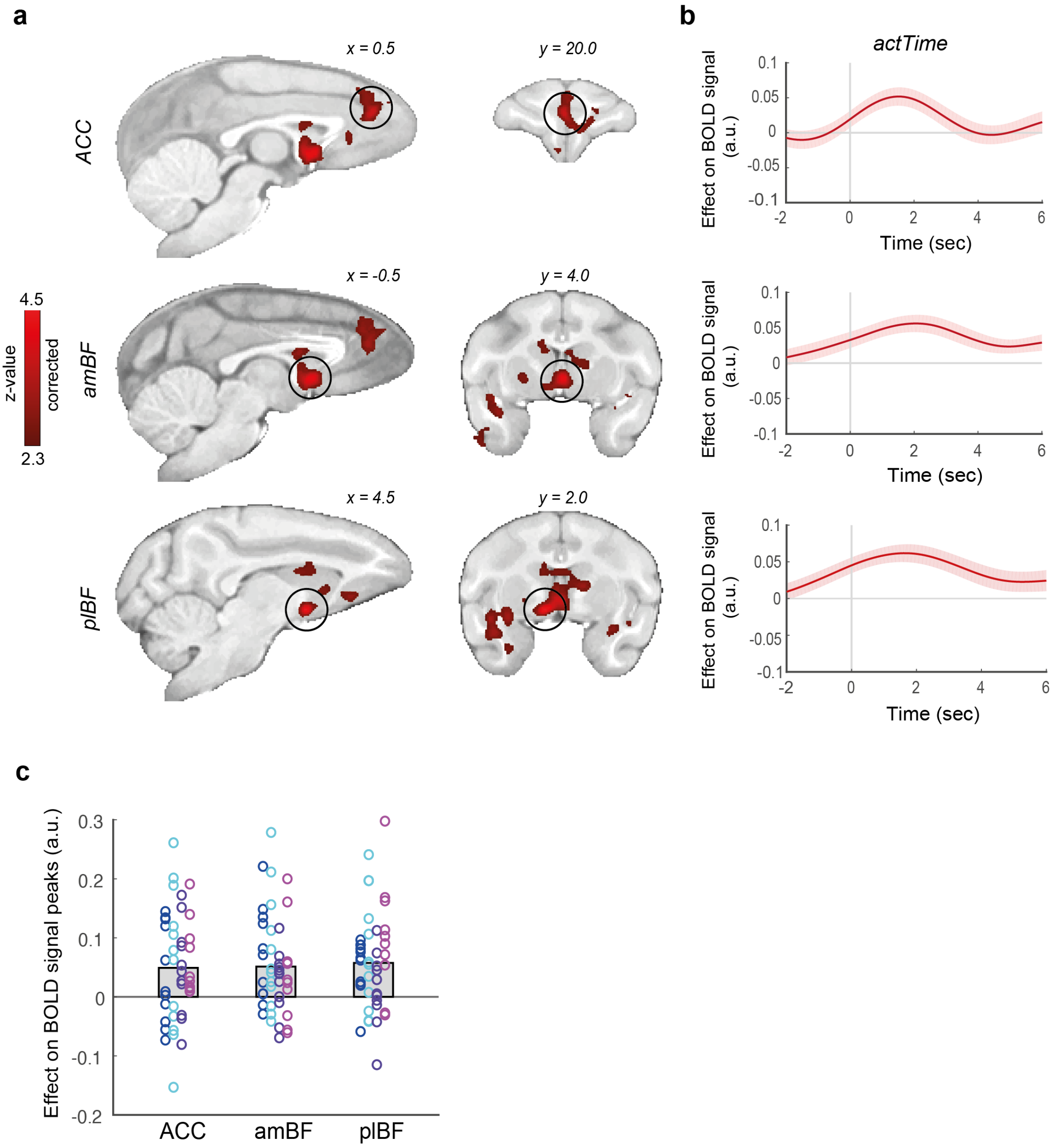
a
x = 0.5
y = 20.0
ACC
x = -0.5
y = 4.0
amBF
4.5
z-value
corrected
2.3
x = 4.5
y = 2.0
plBF
b
actTime
Effect on BOLD signal (a.u.)
Time (sec)
c
Effect on BOLD signal peaks (a.u.)
ACC
amBF
plBF

Figure S2. ACC and BF encode time to act. Related to Figure 4. (a) Whole-brain analysis showing voxels where activity reflected parametric variation in the empirically observed *actTime*, as indexed by dot number at the time of response. The analysis was performed in the same manner as in Fig.4a. but here the GLM only included *actTime* and not all the present and past contextual factors that influenced *actTime*. Top panel; ACC. Middle panel; amBF. Bottom panel; plBF. Whole-brain cluster-based correction, Z>2.3. (b) ROI time-course analysis of the ACC (top panel), amBF (middle panel), and plBF (bottom panel), showing the relationship between BOLD activity and parametric variation in *actTime*, after adding time-to-act in seconds to GLM2.1 (Methods) as a covariate to control for passage of time (Methods, GLM2.2). The lines and shadings show the mean and standard error of the β weights across the sessions, respectively. Time zero is the response time. Note that due to delay in the BOLD hemodynamic response function the BOLD signal time-course peaks 3s after neural activity. Once the delay in BOLD response is taken into account it is clear that BOLD activity reflects neural events occurring before the response onset. (c) No significant difference in *actTime* encoding was observed between ACC, amBF and plBF after adding time-to-act in seconds to the GLM as a covariate to control for passage of time. Each colour represents one animal, and each ring is the peak beta-weight of one testing session. The grey columns illustrate the group mean.

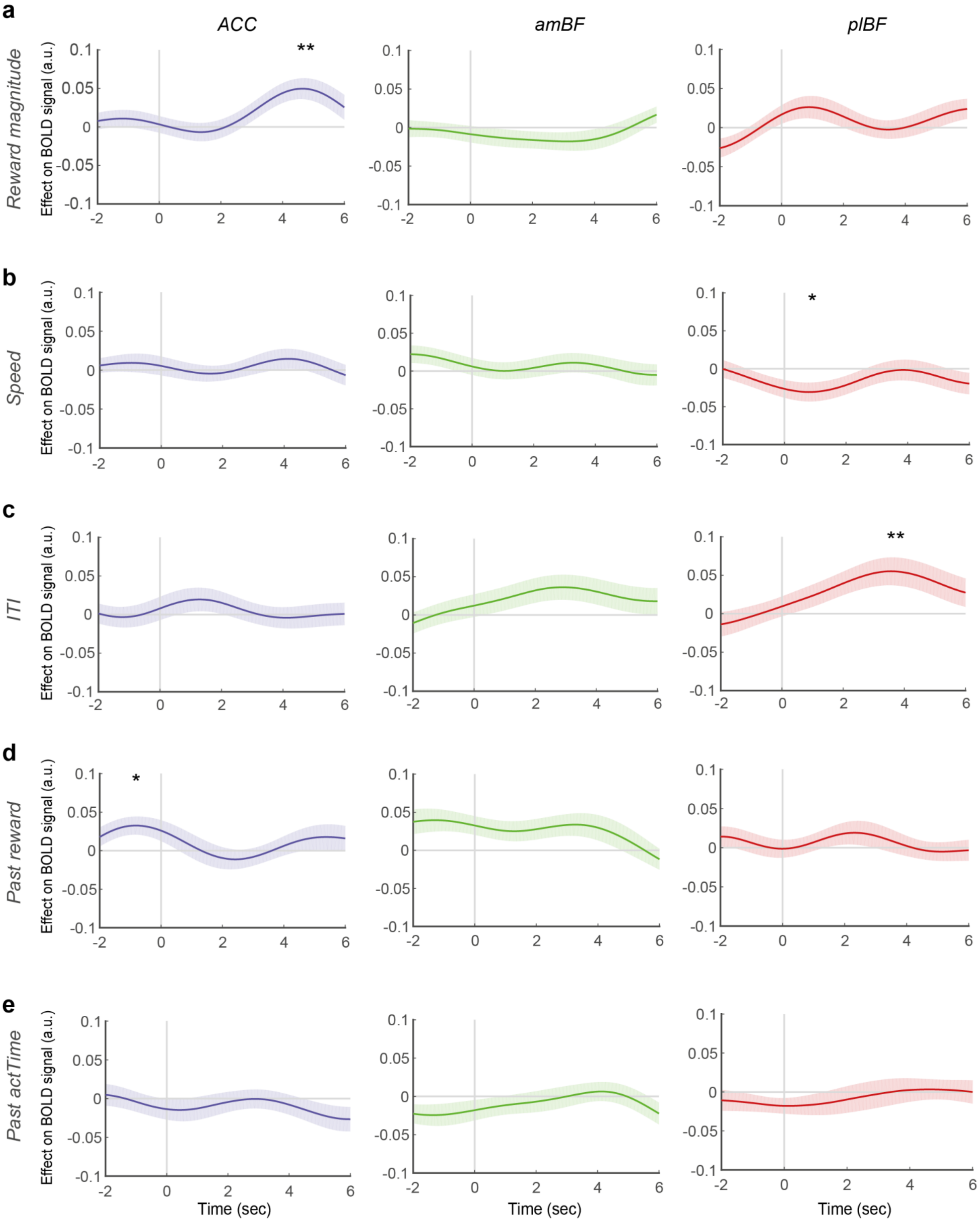

a
b
c
d
e
ACC
amBF
plBF
Reward magnitude
Speed
ITI
Past reward
Past actTime
Effect on BOLD signal (a.u.)
Time (sec)
**
*
**
*

Figure S3. Effect of contextual factors on BOLD signal. Related to Figure 4. ROI time-course analysis of the ACC (blue), amBF (green), and plBF (red), showing the relationship between BOLD activity and parametric variation in reward magnitude (a), dot speed (b), ITI (c), past reward (d), and past *actTime* (e). We found individual effect of contextual factors on ACC (reward magnitude and past reward outcome) and plBF (speed and ITI). Given that both reward magnitude and *actTime* are correlated with the ACC BOLD signal, we asked whether expected value (EV) (the product of reward magnitude and reward probability at the time of response) might be a better predictor of activity in ACC. We performed the same analysis as in GLM2.1 (see methods), however, this time we replaced *actTime* with EV. We found no significant relationship between EV and BOLD signal in ACC ($t(44)=1.19$, $P=0.24$; leave-one-out procedure for peak selection). Importantly, the effect of *actTime* on ACC BOLD signal was stronger than the effect of EV ($t(44)=2.38$, $P=0.02$; paired-samples t-test). ACC activity reflects both reward probability and magnitude and other factors determining EV in choice selection tasks (Cavanagh et al., 2016; Hunt et al., 2015; Kennerley et al., 2009). However, in the current context, in which expected reward magnitude was explicitly cued and action timing was all important and directly under the monkey's control, ACC tracked expected reward magnitude and reflected the *actTime* observed on each trial. *actTime* in turn, albeit non-linearly (via a sigmoid function), determined likelihood of reward. Importantly, a significant effect of individual contextual factors on an ROI signal does not imply that that specific ROI influences the *relationship* between the contextual factor and *actTime*. Instead it simply indicates a direct effect of a contextual factor on ROI BOLD signal, regardless of its influence on *actTime*. We discuss the relationship between contextual factors and *actTime,* and their influence on BOLD signal, later in Figures 5 and 6. The lines and shadings show the mean and standard error of the β weights across the sessions, respectively. Time zero is the response time. Significance testing on time-course data was performed by using a leave-one-out procedure on the group peak signal. One-sample t-tests, * $P<0.05$, ** $P<0.01$, *** $P<0.001$.

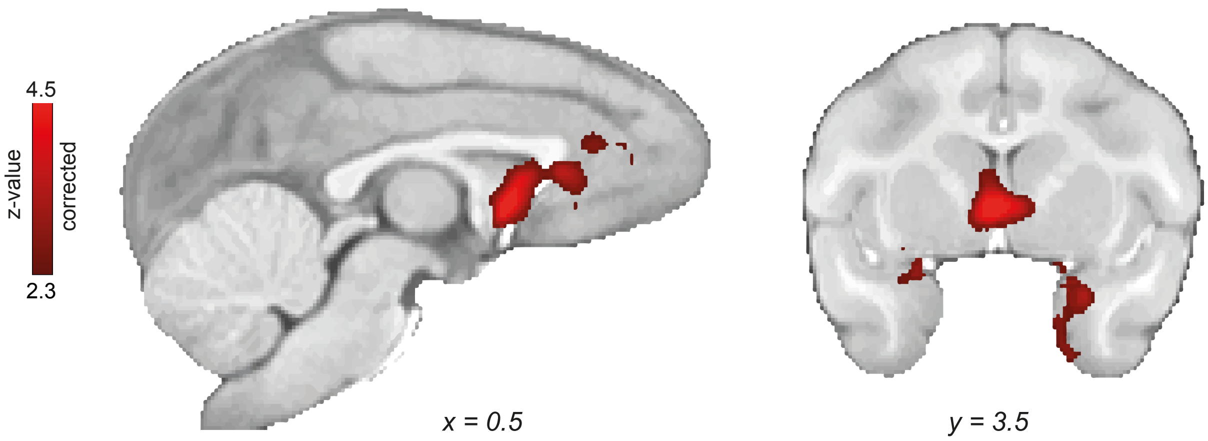

Figure S4. Whole-brain analysis showing voxels where activity reflected parametric variation in the deterministic *actTime*. Related to Figure 5. To verify whether areas outside our ROIs could also encode deterministic *actTime*, we performed a model-based whole-brain analysis. This confirmed that voxels that are correlated with trial-by-trial variation in deterministic *actTime* mainly overlap with the BF ROI. Format as in Fig.S2a, but after replacing observed *actTime* with deterministic *actTime* as predicted by Cox model. Two significant clusters were identified: the peak of the largest cluster was at BF (peak Z=5.1, number of voxels=9203; F99: x=1.0, y=4.0, z=2.0; whole-brain cluster-based correction, Z>2.3, *P*<0.001). The smaller cluster was at the boundary of the left amygdala and entorhinal cortex (peak Z=5.1, number of voxels=2197; F99: x=-9.0, y=4.5, z=-7.0; whole-brain cluster-based correction, Z>2.3, *P*=0.003).

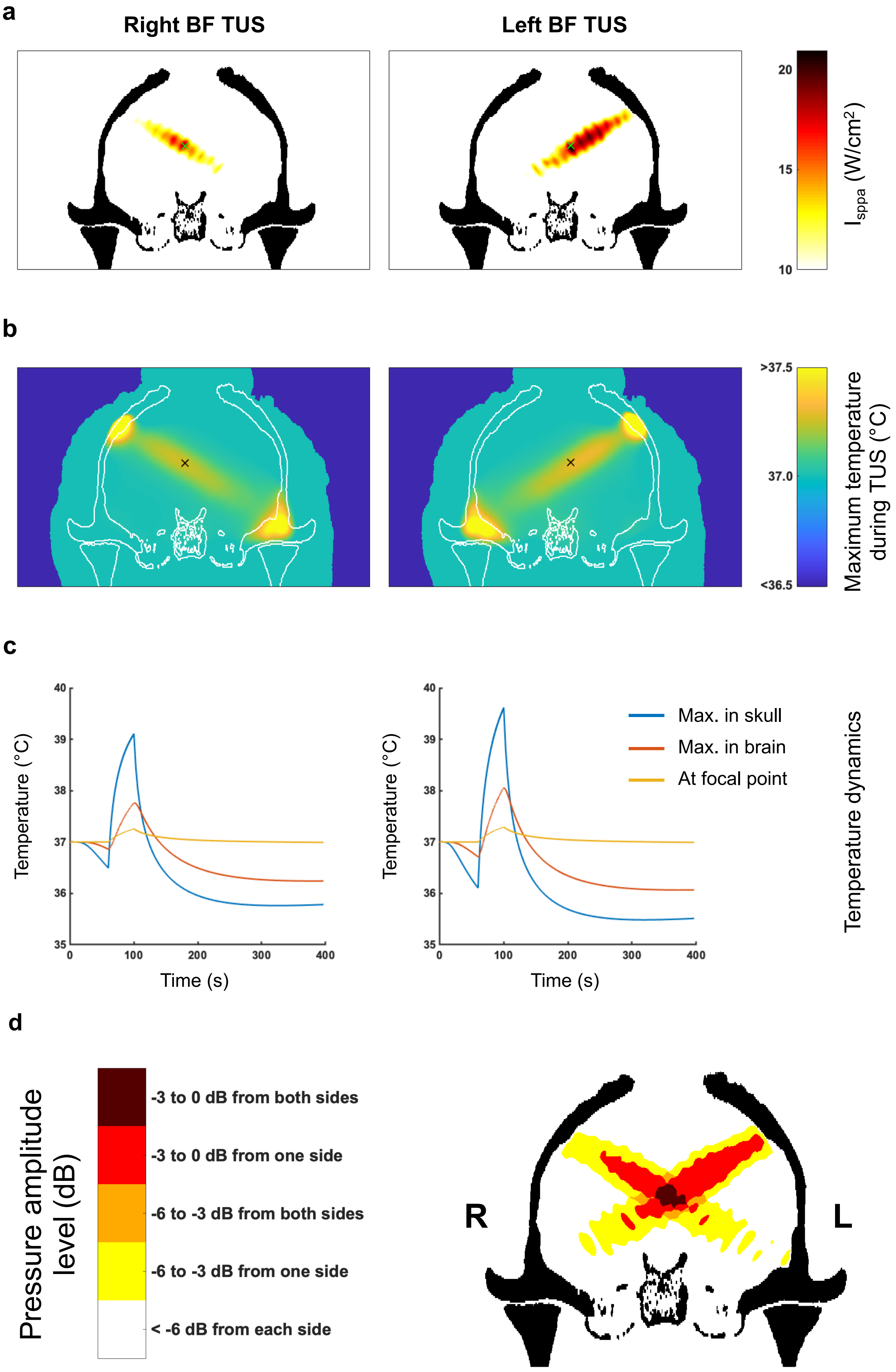

a
Right BF TUS
Left BF TUS
20
15
10
Isppa (W/cm2)
b
>37.5
37.0
<36.5
Maximum temperature during TUS (°C)
c
Temperature (°C)
Time (s)
Max. in skull
Max. in brain
At focal point
Temperature dynamics
d
Pressure amplitude level (dB)
-3 to 0 dB from both sides
-3 to 0 dB from one side
-6 to -3 dB from both sides
-6 to -3 dB from one side
< -6 dB from each side
R
L

Figure S5. Simulation of the acoustic wave propagation and its thermal effect. Related to Figure 7. (a-c) Peak intensities, spatial distribution and thermal modelling for right BF targeting (left column) and left BF targeting (right column). (a) Simulated focused ultrasound peak intensities and spatial distribution in the brain when targeting BF, based on a high-resolution macaque whole-head CT scan. The maximum spatial-peak pulse-averaged intensity (I_{sppa}) at the acoustic focus point was 21.2 W/cm^2 for the left BF target and 18.9 W/cm^2 for the right BF target (spatial peak temporal average intensities, I_{spta}: 6.4 W/cm^2 and 5.6 W/cm^2 for left and right, respectively). (b) Whole-head modelling of the maximum thermal rise during 40 s TUS. BF stimulation target position is shown as a cross for both sides on sagittal views ((a) and (b)). (c) Temperature dynamics for the hottest point in the skull (blue), the hottest point in the brain (red) and the geometrical focal point in the brain (yellow). Given that the skull is more acoustically absorbing than soft tissue, the highest thermal increase is located in the skull itself, estimated by the simulation to be 2.6°C and 2.1°C for left and right, respectively. For an approximate 0.5 mm thickness of the dura the maximum temperature in the brain below the dura was 38.1°C and 37.8°C for left and right, respectively. The maximal thermal increase at the geometrical focus of the sonic transducer was less than 0.3 °C for both sides. (d) Representation of pressure amplitude overlap from successive left and right BF TUS. Pressure fields were obtained from whole-head simulations of the focused ultrasound pressure amplitude when targeting left and right BF, based on a high-resolution macaque whole-head CT scan. The pressure fields are superimposed and are represented in relative pressure levels where 0 dB is the maximum pressure amplitude estimated in the brain. Dark red and orange highlight areas where the pressure is higher than -3dB and -6dB, respectively, for both sonications due to an overlap of the two beams. Red and yellow highlight areas where the pressure is higher than -3dB and -6dB, respectively, for one of the sonications.

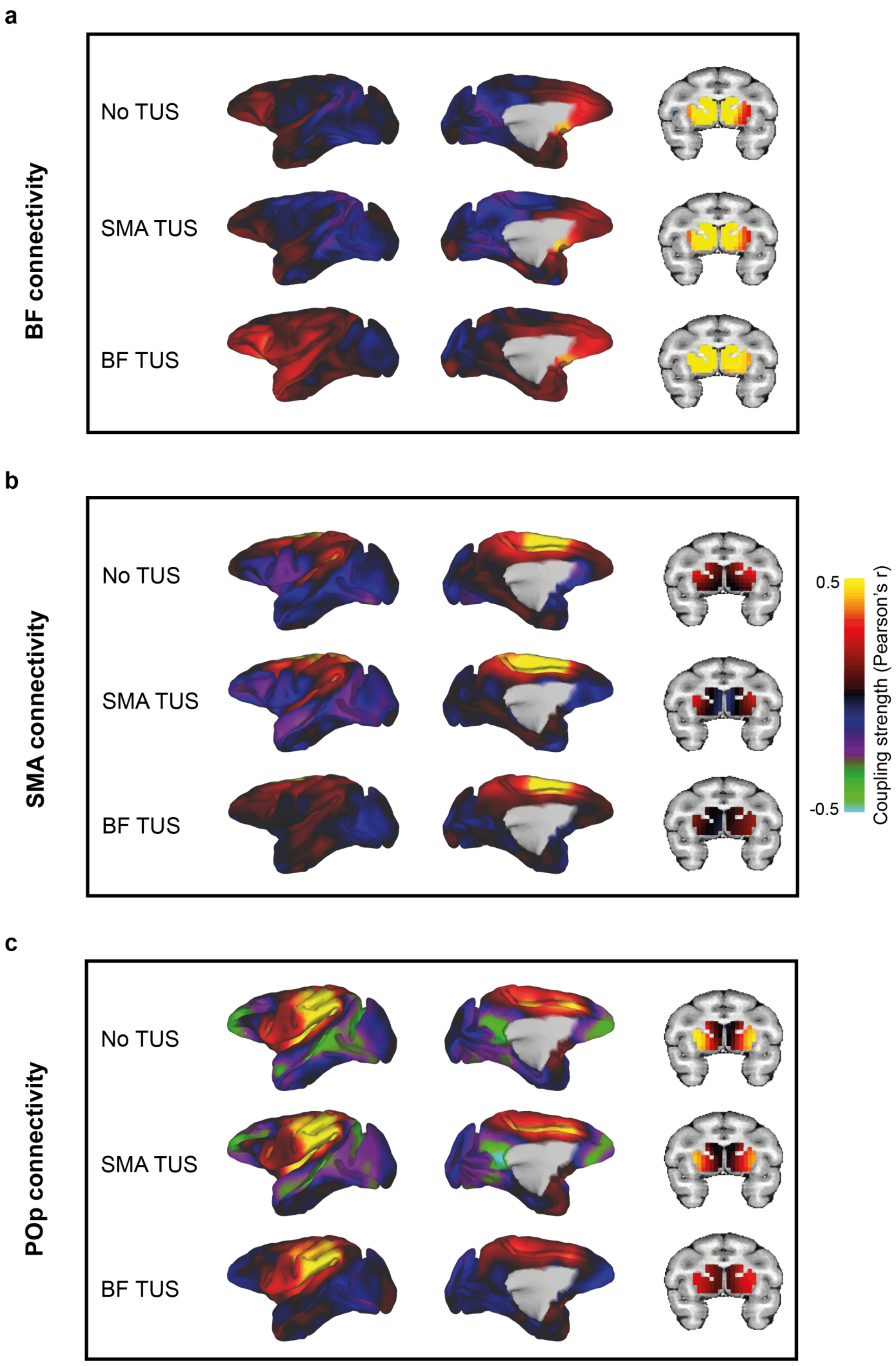
a
BF connectivity
No TUS
SMA TUS
BF TUS
b
SMA connectivity
No TUS
SMA TUS
BF TUS
0.5
-0.5
Coupling strength (Pearson's r)
c
POp connectivity
No TUS
SMA TUS
BF TUS

Figure S6. Effects of TUS were specific to stimulation of BF. Related to Figure 7.

Whole-brain functional connectivity of the BF (a), a control region in the SMA (b), and a control region in POp (c). In each panel the upper row shows functional connectivity from the seed area to the rest of the brain without applying TUS. The middle and bottom rows show seed-based connectivity after SMA and BF TUS, respectively. Positive correlations are represented in warm colours from red to yellow, negative correlations are represented in cool colours from blue to green. After TUS over BF, BF positive coupling (a) is enhanced within BF and between BF and frontal and cingulate gyri. Importantly, this pattern is not observed when looking at the connectivity between SMA (b) or POp (c) and the rest of the brain. In fact, panels b and c show a widespread suppression of coupling elsewhere in the brain after BF TUS, compared to no TUS and SMA TUS.

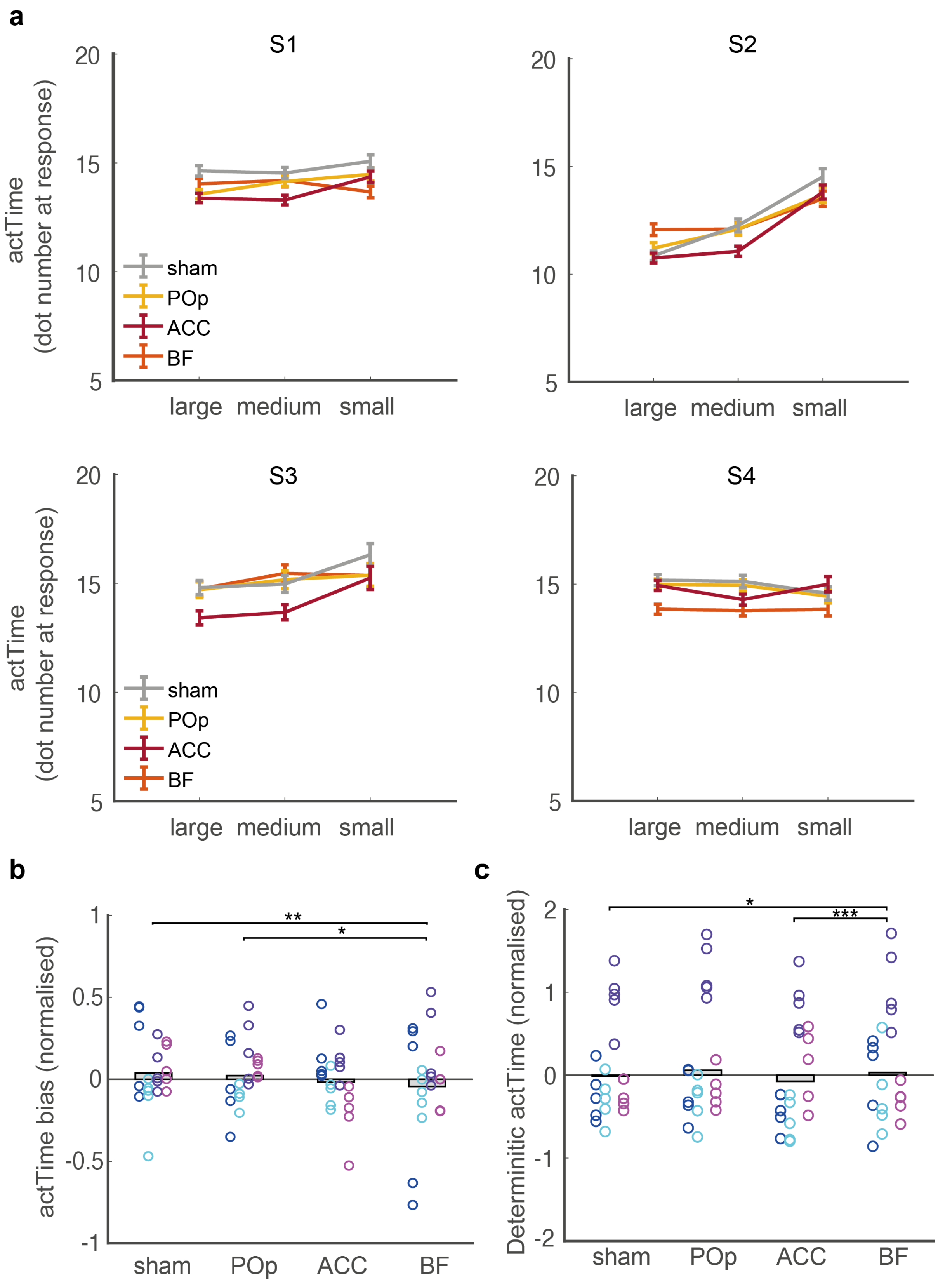

a
S1
S2
S3
S4
actTime
(dot number at response)
sham
POp
ACC
BF
large
medium
small
20
15
10
5
b
c
actTime bias (normalised)
Determinitic actTime (normalised)
sham
POp
ACC
BF
**
*
***
1
0.5
0
-0.5
-1
2
-2

Figure S7. Behavioural effects of TUS. Related to Figure 7. (a) Animals acted quicker after ACC than after BF, POp or sham TUS, when offered medium or large, compared to small rewards. Format as in Fig.7di but with data from each animal (S1, S2, S3, S4) reported separately. Error bars show standard error of the mean. (b-c) The effect of BF TUS on *actTime bias* (b) and deterministic *actTime* (c). *actTime bias* was defined as the trial-by-trial absolute difference between observed and deterministic *actTime*. We found a statistically significant effect of TUS on *actTime bias,* however, careful inspection of the data shows that the effect is not consistent across all four animals. We could not include by-subject random slope in the model as it failed to converge due to our small sample size (n=4). Further analyses of each individual animal's data revealed that in two animals there was a significant main effect of TUS location on *actTime bias* (S1, $X^2(3)=15.94$, $P=0.001$; S4, $X^2(3)=14.86$, $P=0.002$) and in one animal there was a significant interaction between the effect of TUS location and time (the effect of BF TUS became more marked as the session progressed) (S2, $X^2(3)=10.54$, $P=0.015$). The effect was not significant in the fourth animal (S3). Format as in Fig.7e but with data from each animal and each stimulation session reported separately. Each colour represents one animal, and each ring is one stimulation session. The black grey columns illustrate the group mean across all observations. Multilevel ANOVA followed by pairwise t-test. * $P<0.05$, ** $P<0.01$, *** $P<0.001$.

Cluster	P	Z-max	Z-max X (mm)	Z-max Y (mm)	Z-max Z (mm)	COPE-mean
BF	0.008	4.5	4.5	2.0	-2.0	54.1
ACC	0.004	3.9	0.5	20.5	12.5	54.8
Right anterior insula	<0.001	3.9	19.6	3.0	-5.0	49.7
Left putamen	< 0.001	3.9	-10.5	-1.5	5.0	45.1

Table S1. List of all clusters. Related to Figure 4. We found four significant clusters at threshold Z > 2.3. In this study we focused on BF and ACC with bilateral/midline activity.
