# Supplementary material for: A Basal Forebrain-Cingulate Circuit in Macaques Decides It Is Time to Act
Source: Neuron. 2020 Jan 22;105(2):370–384.e8. doi: 10.1016/j.neuron.2019.10.030 (PMC6975166; doi:10.1016/j.neuron.2019.10.030)
Supplement: Document S2. Article plus Supplemental Information [file mmc2.pdf]

Article

Neuron

A Basal Forebrain-Cingulate Circuit in Macaques Decides It Is Time to Act

Highlights

- Likelihood and timing of voluntary action in macaques can be partially predicted
- Recent experience and present context influence when voluntary action occurs
- A basal forebrain-cingulate circuit mediated effects of these factors on behavior
- Stimulation of this circuit by ultrasound changed decisions about when to act

Authors

Nima Khalighinejad, Alessandro Bongioanni, Lennart Verhagen, ..., Jean-Francois Aubry, Jerome Sallet, Matthew F.S. Rushworth

Correspondence

nima.khalighinejad@psy.ox.ac.uk

In Brief

Deciding when to act is crucial for animals' survival. Khalighinejad et al. used neuroimaging and ultrasound to identify a basal forebrain-cingulate circuit that tracks contextual factors in animals' environments and mediates their effects on animals' decisions about when to act.

Khalighinejad et al., 2020, Neuron *105*, 370–384
January 22, 2020 © 2019 The Author(s). Published by Elsevier Inc.
https://doi.org/10.1016/j.neuron.2019.10.030

Neuron
Article

A Basal Forebrain-Cingulate Circuit in Macaques Decides It Is Time to Act

Nima Khalighinejad,[1,6,*] Alessandro Bongioanni,[1] Lennart Verhagen,[1,2] Davide Folloni,[1] David Attali,[3,4,5] Jean-Francois Aubry,[3] Jerome Sallet,[1] and Matthew F.S. Rushworth[1]
[1]Wellcome Centre for Integrative Neuroimaging, Department of Experimental Psychology, University of Oxford, Oxford OX1 3SR, UK
[2]Donders Institute for Brain, Cognition and Behaviour, Radboud University Nijmegen, Nijmegen 6525 XZ, the Netherlands
[3]Physics for Medicine Paris, INSERM U1273, ESPCI Paris, CNRS FRE 2031, PSL Research University, Paris 75012, France
[4]Pathophysiology of Psychiatric Disorders Laboratory, Inserm U1266, Institute of Psychiatry and Neuroscience of Paris, Paris Descartes University, Paris University, Paris 75014, France
[5]Service Hospitalo-Universitaire, Sainte-Anne Hospital, UGH Paris Psychiatry and Neurosciences, Paris 75014, France
[6]Lead Contact
*Correspondence: nima.khalighinejad@psy.ox.ac.uk
https://doi.org/10.1016/j.neuron.2019.10.030

SUMMARY

The medial frontal cortex has been linked to voluntary action, but an explanation of why decisions to act emerge at particular points in time has been lacking. We show that, in macaques, decisions about whether and when to act are predicted by a set of features defining the animal's current and past context; for example, respectively, cues indicating the current average rate of reward and recent previous voluntary action decisions. We show that activity in two brain areas—the anterior cingulate cortex and basal forebrain—tracks these contextual factors and mediates their effects on behavior in distinct ways. We use focused transcranial ultrasound to selectively and effectively stimulate deep in the brain, even as deep as the basal forebrain, and demonstrate that alteration of activity in the two areas changes decisions about when to act.

INTRODUCTION

Leopards are expert stalkers. When they are in close proximity of prey, they ambush and wait for the right moment. If they charge too early, then the element of surprise is lost, and the prey will run away. If they charge too late, then they risk being detected. Deciding when to lunge is crucial for their survival. This decision not only comprises when to act but entails a decision regarding whether it is worth acting at all; there may be situations in which the prey is simply not worth pursuing or times when it is necessary to repress the urge to act on immediate desires. Deciding requires integrating information from the surrounding environment with past hunting experiences and internal state. In the current experiment, we examine precisely this question: how the current environmental context and recently experienced past contexts combine to influence "voluntary" decisions about when to act. We identify and record neural activity mediating decisions about when to act and examine the effect of manipulating the activity using ultrasound stimulation.

We introduced a new paradigm to investigate, in the macaque, how contextual factors and internal state, shaped by the present and past environment, are integrated to influence whether and when to act. Four macaques were trained to track the number of dots on a screen while in the MRI scanner. Dots appeared one at a time on a screen, and animals could decide to make a response, at a time of their choice, by tapping on a response pad in front of them (Figures 1A and 1B). The number of dots on the screen at the time of response determined the probability of reward. Reward probability was drawn from a sigmoid function; the longer the animals waited before responding, the more dots appeared on the screen, and the higher was the probability of reward (Figure 1D). Impulsive responses were unlikely to yield a reward. The probability distribution remained constant across the trials and sessions. Three features determined the present context: reward magnitude, the speed of the sequential appearance of the dots, and the inter-trial interval (ITI). Different levels of reward magnitude and ITI were associated with different dot colors and patterns, respectively (Figure 1C). These "present contextual factors" were varied independently of one another and in a pseudo-randomized order (Figure 1E). The reward magnitude and dot speed varied from trial to trial, and the ITI varied in blocks of 30 trials. In addition to the present context, the past context also varied. The past context was defined by the animal's own recent behavior and recent reward experience—the outcomes and action times of recent past trials (Figure 1C).

First, behavioral analyses demonstrated that both types of contextual information influenced decisions about whether and when to act. A large proportion of variance in decisions about when to act could be explained by a quantitative model that deduced a deterministic component of time to act by identifying features of the environment relating to both the current context and the recent past context. Second, we used fMRI to look for brain activity that is parametrically related to the factors that change the likelihood of action rather than action initiation per se. We identified two areas—the anterior cingulate cortex (ACC) and basal forebrain (BF)—that tracked these contextual factors and mediated their effect on behavior in distinct ways. Third, we used focused transcranial ultrasound stimulation (TUS) to modulate activity in these brain areas. We simulated the acoustic wave propagation and obtained offline resting-state

 © 2019 The Author(s). Published by Elsevier Inc.
This is an open access article under the CC BY license (http://creativecommons.org/licenses/by/4.0/).

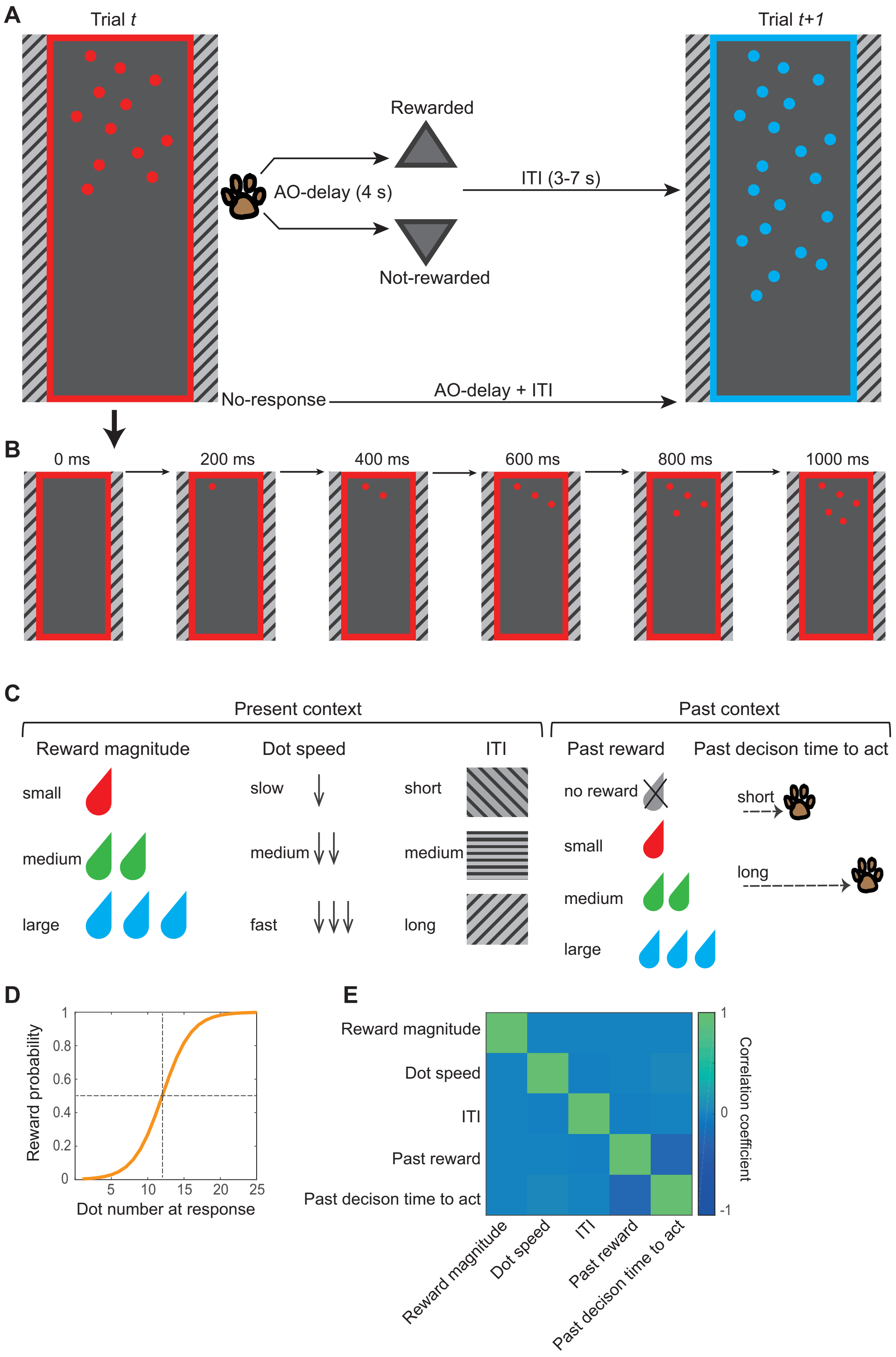

(legend on next page)

fMRI (rs-fMRI) to show that it is possible to manipulate activity in an area deep in the brain, such as the BF, using TUS. Fourth, we showed that alteration of activity in the ACC and BF by TUS changed decisions about when to act.

RESULTS

Animals Used Contextual Factors to Decide Whether and When to Act

We used a hierarchical linear model to test whether animals use contextual factors to decide when to act. Time to act (actTime) was indexed by the number of dots on the screen at the time of response. Note that there was a correspondence (a sigmoid relationship; Figure 1D) between dot number and reward probability and that experimental manipulation of "dot speed" ensured that actTime as indexed by the number of dots was decorrelated from time measured in seconds. This facilitated interpretation of the results; changes in actTime, as measured by number of dots, therefore signified a deliberate decision process rather than merely passage of time. On average, animals waited for 14 ± 4 dots before responding (Figure S1A), which was associated with a 73% chance of reward. Given the sigmoid distribution of reward function, there is little to gain by employing an actTime of longer than 17 dots (92% chance of reward; Figure 1D). The task finished after 40 min regardless of the number of trials performed. That means that animals might collect fewer rewards overall across the whole session when they waited longer before responding on any given trial. A multilevel ANOVA (STAR Methods) showed that all aspects of present context (reward magnitude, $\chi^2(2) = 284$, $p < 0.001$; dot speed, $\chi^2(2) = 1{,}465$, $p < 0.001$; and ITI, $\chi^2(2) = 44$, $p < 0.001$) and past context (reward outcome on past trial, $\chi^2(3) = 144$, $p < 0.001$; and actTime on past trial, $\chi^2(1) = 25$, $p < 0.001$) influenced animals' actTime. actTime was longer during long compared with short ITI blocks (Tukey's honestly significant difference [HSD]; $\beta = 0.16 \pm 0.02$, $Z = 6$, $p < 0.001$) (Figure 2A), under fast compared with slow dot speed conditions ($\beta = 1.06 \pm 0.02$, $Z = 42$, $p < 0.001$) (Figure 2B), when offered a large compared with a medium reward ($\beta = 0.22 \pm 0.03$, $Z = 9$, $p < 0.001$), and when offered a small compared with a medium reward ($\beta = 0.44 \pm 0.03$, $Z = 17$, $p < 0.001$), giving rise to a U-shaped effect of reward magnitude on actTime (Figure 2C; see Figure S1 for further discussion). actTime was shorter when they had received a large reward compared with no reward on the past trial ($\beta = -0.36 \pm 0.03$, $Z = -11$, $p < 0.001$; Figure 2D), and it was longer when they had already delayed actTime on the past trial ($\beta = 0.07 \pm 0.01$; Figure 2E).

Having found an effect of contextual factors on decisions about when to act, we asked whether the same factors influence whether it is worth acting as well as how quickly to do so. We used hierarchical logistic regression to predict the odds of responding from present and past contextual factors and showed that this was indeed the case, confirming the importance of these factors in influencing self-initiated action (Figure 2F). Across all testing sessions, animals refrained from responding in 16.38% ± 11.42% of the trials. They were more likely to respond in large and medium compared with small reward trials (large versus small, odds ratio [OR] = 12.82, $Z = 17.92$, $p < 0.001$; medium versus small, OR = 6.94, $Z = 16.58$, $p < 0.001$) and when they had been rewarded on the past trial (large versus no reward, OR = 3.27, $Z = 7.58$, $p < 0.001$; medium versus no reward, OR = 2.10, $Z = 4.93$, $p < 0.001$; small versus no reward, OR = 2.57, $Z = 5.66$, $p < 0.001$). On the other hand, they were more likely to refrain from responding in long ITI blocks (long versus short, OR = 0.48, $Z = -6.23$, $p < 0.001$) and when they had waited for a long time on the past trial (OR = 0.86, $Z = -2.55$, $p = 0.01$). These results suggest that animals were less likely to respond when the average reward rate of the environment was low.

It is not clear why animals sometimes refrain from responding in an experimental task when any task-related response is usually likely to increase the likelihood of reward. Nevertheless, it is well known that they often do so (San-Galli et al., 2018; Stoll et al., 2016a). One way to interpret the results is that the animals doing so are avoiding paying a cost that is entailed by performing the trial. Performing a trial requires more than just the motor act of responding and also entails cognitive demands related to stimulus attention and response withholding (Manohar et al., 2015). This might mean that animals may judge the effort of engaging with a trial as not worth the reward that could be obtained for doing so when the reward rate is low.

Contextual Factors Explained a Large Proportion of Variance in Time to Act

To determine the fraction of actTime that was explainable by present and past contextual factors, we used a Cox proportional

Figure 1. Experimental Task

(A) On each trial, animals tracked the number of dots on the screen (maximum number of dots = 25). They could make a response, at a time of their own choice, by touching a response pad in front of them. If they responded, then they received drops of juice or no juice as a function of the reward probability distribution at the time of response. There was a 4-s delay between the response and the outcome (AO-delay). Successful and unsuccessful outcomes are indicated by an upward- and downward-pointing triangle, respectively. The triangle remained on the screen for 2 s. If rewarded, then drops of juice were delivered by a spout near the animal's mouth. The outcome phase was followed by an inter-trial interval (ITI; 3–7 s). If animals did not respond by 300 ms after the last dot, then the frame disappeared, and they had to wait for an AO-delay + ITI before the next trial (trial $t+1$) started. The color of the frame and the dots represent the potential reward magnitude on that trial. The patterns on each side of the screen represent the duration of the ITI.

(B) Timeline of one trial. At the beginning of each trial, an empty frame appeared on the left or right side of the screen. The frame was gradually filled with dots emerging from top to bottom. The dots appeared every 100, 200, or 300 ms, depending on trial type (in the example shown, a new dot emerges every 200 ms).

(C) Contextual factors from current and past trials were used to predict animals' actTime. Present contextual factors consisted of reward magnitude (three levels), dot speed (three levels), and ITI (three levels). They were varied independently of one another and in a pseudo-randomized order. Reward and dot speed changed from trial to trial. The ITI changed in blocks of 30 trials. Past contextual factors consisted of reward outcome (four levels) and actTime on the past trial (continuous variable).

(D) The probability of getting a reward increased as more dots appeared on the screen, following a sigmoid curve. The probability distribution was constant across the trials and sessions.

(E) Correlation matrix of regressors from fMRI design.

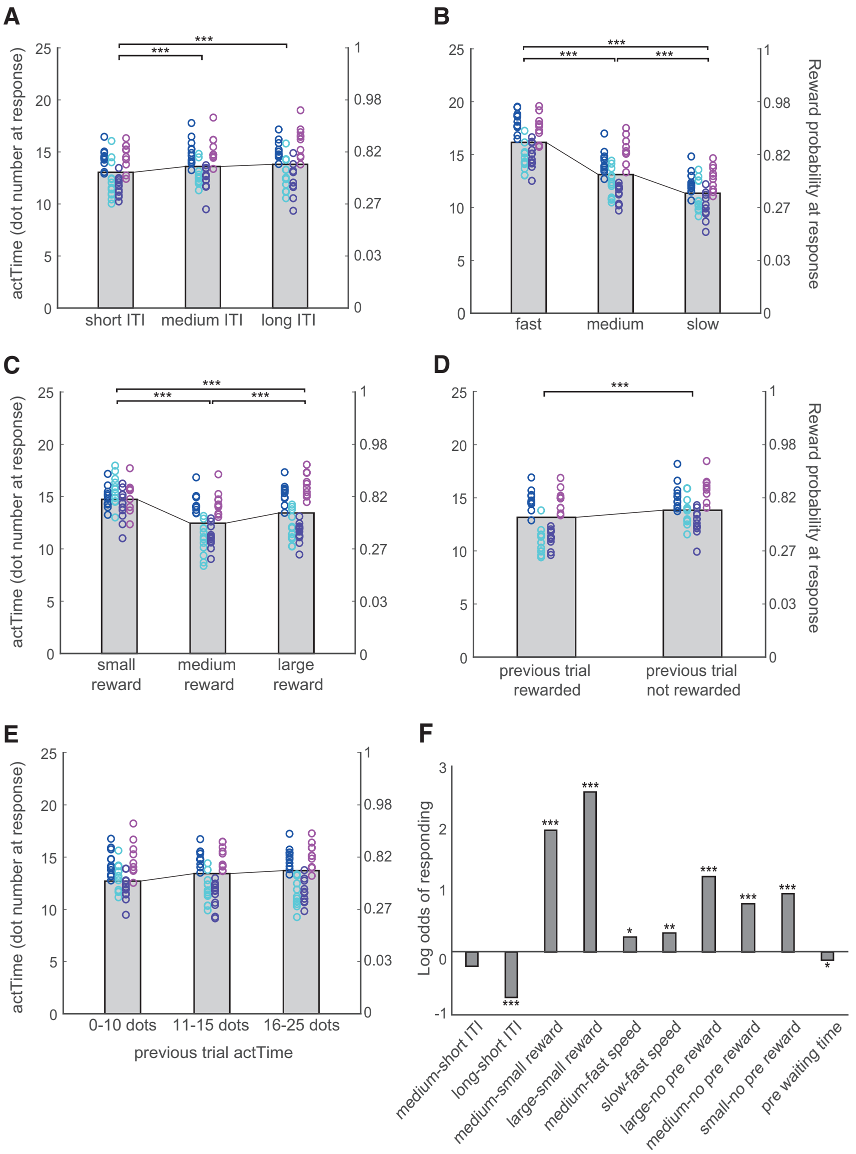

Figure 2. Animals Used Contextual Factors to Decide Whether and When to Act

(A–E) The effect of present and past contextual factors on actTime (left y axis). The corresponding reward probability at response is displayed on the right y axis. actTime was longer in long (7 s) and medium (5 s) compared with short (3 s) ITI blocks (A); in fast compared with slow trials (B); when they were offered a large compared with a medium reward and when offered a small compared with a medium reward, giving rise to a U-shaped effect of reward magnitude on actTime (C); when a previous trial was not rewarded (D); and when they had already delayed actTime on the past trial (E). For illustrative purposes, actTime in the immediate past trial is binned into three groups. Each color represents one animal, and each ring is one testing session. The gray columns illustrate the group mean across all observations. Multilevel ANOVA followed by Tukey's HSD.

(F) The log of the odds of responding during the trial for different levels of present and past contextual factors.

*$p < 0.05$, **$p < 0.01$, ***$p < 0.001$.

See also Figure S1.

hazards model. Cox regressions are a class of survival models suitable for relating the time that passes before a specific event to one or more covariates. This model has been used previously to estimate action time in rats (Murakami et al., 2017), although here we expand the approach to consider the influence of a wider range of factors that reflect both past and present context on actTime (STAR Methods).

First we asked whether present and past contextual factors significantly contribute to the model (all subsequent tests are corrected for multiple comparisons; Figure 3A). The Cox regression coefficients were significantly negative for dot speed (one-sample t test; $t(44) = -15.5$, $p < 0.001$), ITI ($t(44) = -5.5$, $p < 0.001$), and actTime on past trials (significant in 4 of 10 past trials; $t(44) < -3.72$, $p < 0.02$), suggesting that slower dot speed and longer ITI on the current trial and longer actTime on past trials lengthen actTime on the current trial. Cox regression coefficients were significantly positive for expected reward magnitude on the current trial ($t(44) = 8$, $p < 0.001$) and reward outcome on the past trials (significant for the trial immediately preceding the current trial; $t(44) = 12.38$, $p < 0.001$); a larger potential reward on the current trial and a larger reward outcome on the past trial shorten actTime on the current trial. We then used the Cox regression coefficients from the current and immediately preceding trial (only coefficients from the immediately preceding trial were used because only these had been significant for both past reward outcome and past actTime) to estimate the expected actTime at each trial (STAR Methods; Figure 3B). We termed this estimate deterministic $actTime_{present + past\ context}$, which is defined as the number of dots, given present and past context, at which an animal is expected to respond on any given trial. Deterministic actTime was also later used for model-based fMRI analysis.

Subsequently, we estimated the Cox regression coefficients separately from their present and past components. We then used the Cox coefficients relating to either the present or the immediately preceding trial to derive two separate actTime estimates. These new estimates were termed deterministic $actTime_{present\ context}$ (Figure 3C) and deterministic $actTime_{past\ context}$ (Figure 3D), respectively. Finally, we asked what percentage of variability in observed actTime could be explained by present, past, or a combination of both contexts (STAR Methods). On average, present and past contextual factors together explained 36% ± 9% of actTime variance. Of these, 25% ± 10% and 12% ± 5% were explained by present and past contextual factors, respectively (Figure 3E).

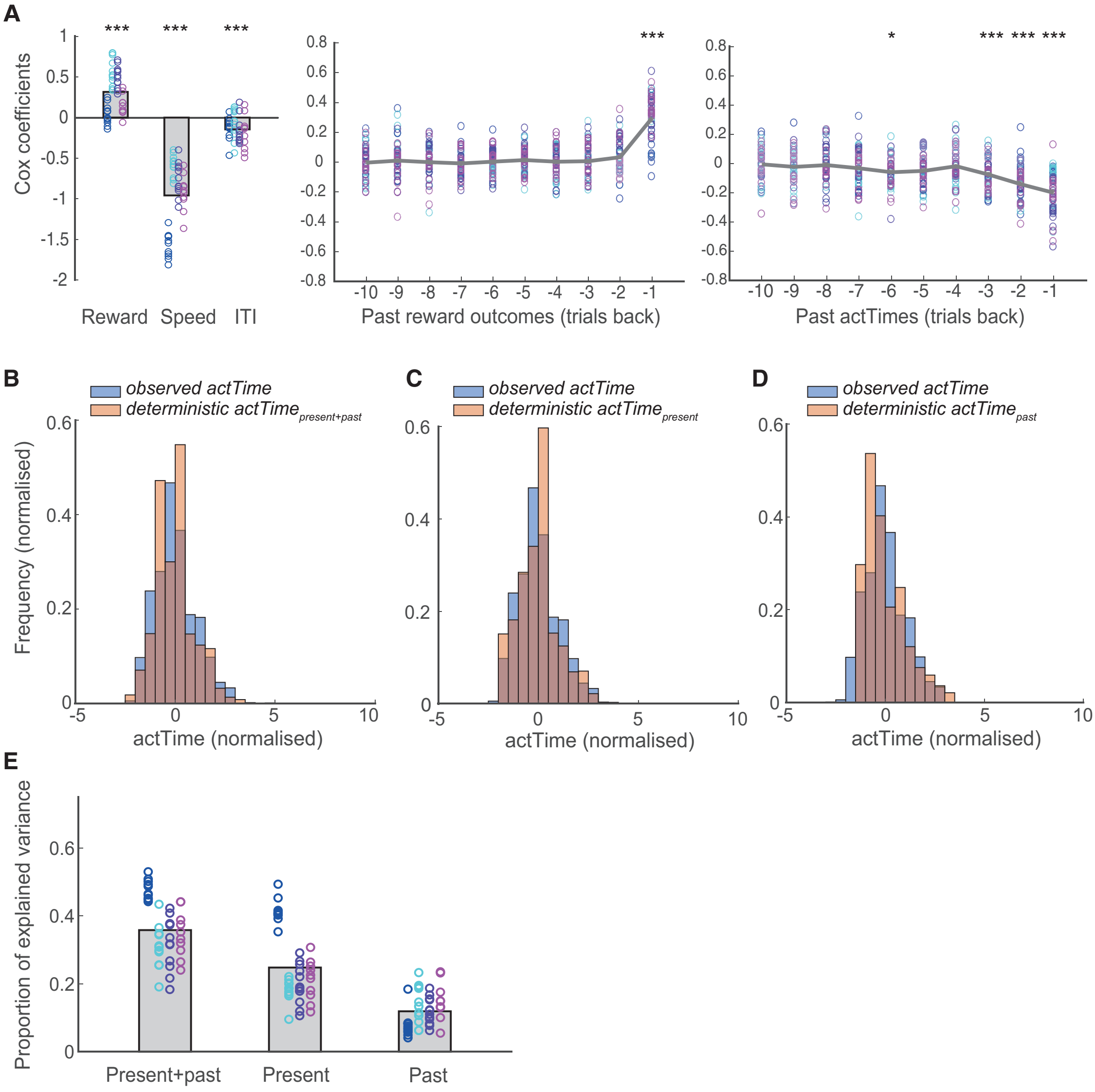

Figure 3. Contextual Factors Explained a Large Proportion of Variance in Time to Act

(A) Cox regression coefficients for present (left panel) and past (middle and right panels) contextual factors. A negative Cox coefficient means a negative effect of predictor on the probability of responding.

(B–D) Cox regression coefficients were used to estimate deterministic actTime on each trial. Deterministic actTime is superimposed on observed actTime for comparison. Deterministic actTime was estimated from both present and past contextual factors (B), from present contextual factors alone (C), and from past contextual factors alone (D).

(E) Proportion of variance (PEV) in actTime explained by the Cox regression model. PEV is estimated separately from present and past, present, and past contextual factors. Each color represents one animal, and each ring is one testing session. The gray columns illustrate the group mean.

One-sample t tests; *p < 0.05, **p < 0.01, ***p < 0.001, corrected for multiple comparisons.

ACC and BF Activity Is Correlated with Time to Act

Having shown that animals use contextual factors to decide when to act, we used fMRI to identify potential brain mechanisms mediating this behavior. We first used a generalized linear model (GLM; STAR Methods, GLM.1) to look for brain areas in which activity reflected parametric variation in the empirically observed actTime, as indexed by dot number at the time of response, and then asked whether the same areas integrated contextual factors to compute deterministic actTime derived from the Cox model. The first analysis revealed two main bilateral/midline regions in which a blood-oxygen-level-dependent (BOLD) signal was modulated by observed actTime (whole-brain cluster-based correction, $Z > 2.3$, $p < 0.01$; Table S1): (1) ACC (peak $Z = 3.88$, Caret-F99 Atlas [F99]: x = 0.5, y = 20.5, z = 12.5), (2) BF. The activity in the BF extends from the anterior-medial BF (amBF) containing the medial septum/diagonal band of Broca (peak $Z = 4.34$, F99: x = −0.5, y = 4.0, z = 1.0) to the posterior-lateral BF (plBF) containing the *nucleus basalis* of Meynert (peak $Z = 4.49$, F99: x = 4.5, y = 2.0,

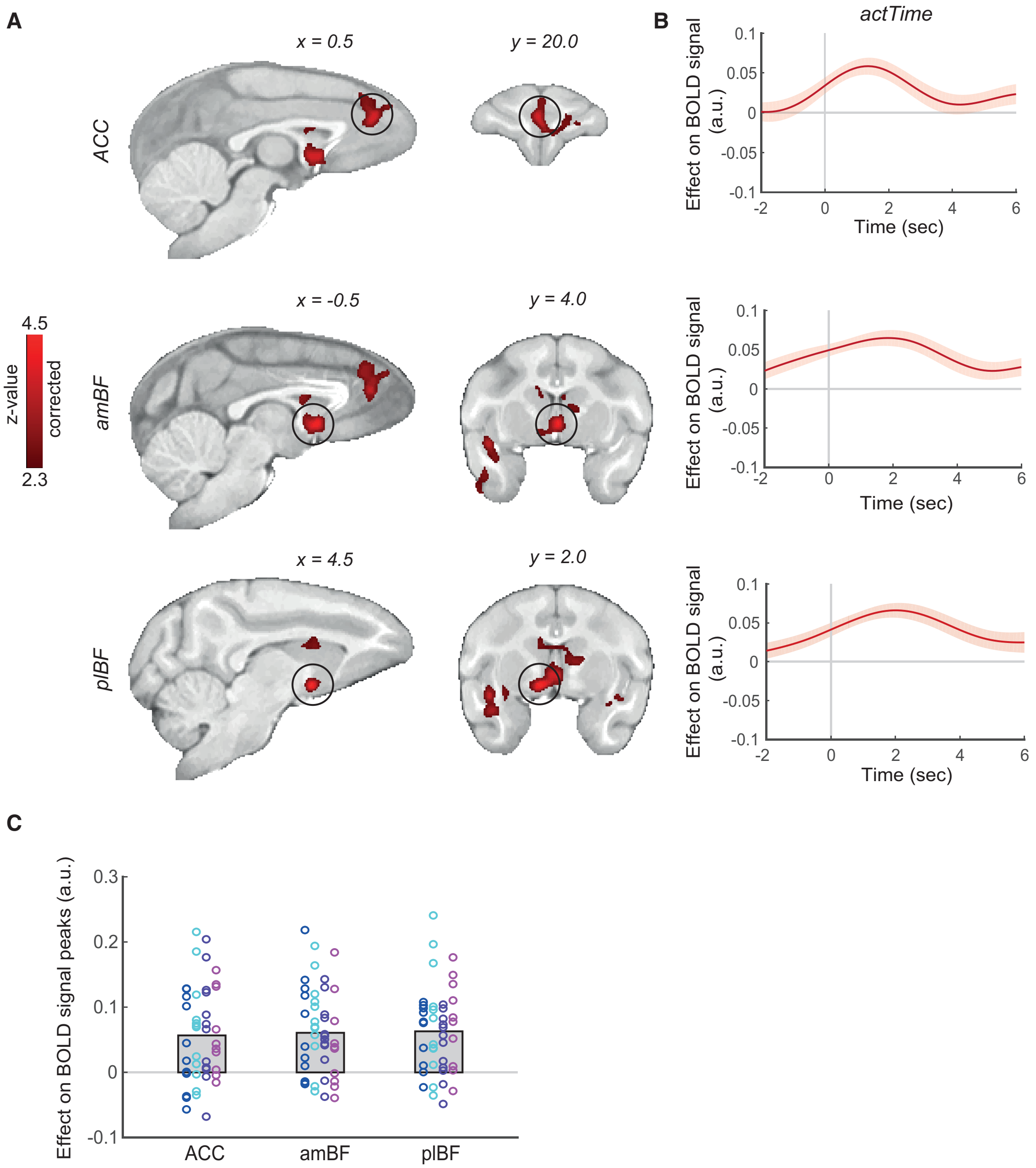

Figure 4. The ACC and BF Encode Time to Act

(A) Whole-brain analysis showing voxels where activity reflected parametric variation in the empirically observed actTime. Here we focused on areas with bilateral/midline activity: the ACC (top panel) and BF (middle [amBF] and bottom [plBF] panels). Whole-brain cluster-based correction, $Z > 2.3$.

(B) ROI time course analysis of the ACC (top panel), amBF (middle panel), and plBF (bottom panel), showing the relationship between BOLD and actTime. The lines and shadings show the mean and standard error (SE) of the β weights across the sessions, respectively. Time zero is the response time. Note that, because of delay in the BOLD hemodynamic response function, the BOLD signal time course peaks 3 s after neural activity. When the delay in BOLD response is taken into account, it is clear that BOLD activity reflects neural events occurring before the response onset.

(C) No significant difference in actTime encoding was observed between the ACC, amBF, and plBF. Each color represents one animal, and each ring is the peak beta-weight of one testing session. The gray columns illustrate the group mean.

See also Figures S2 and S3 and Table S1.

$z = -2.0$) (Figure 4A; see Figure S2A for an alternative analysis). Corresponding BF sub-regions have been described in the human BF (Fritz et al., 2019; Markello et al., 2018).

To illustrate the timing of encoding of observed actTime in the ACC, amBF, and plBF, we extracted and averaged the BOLD time course of each voxel within each region of interest (ROI)

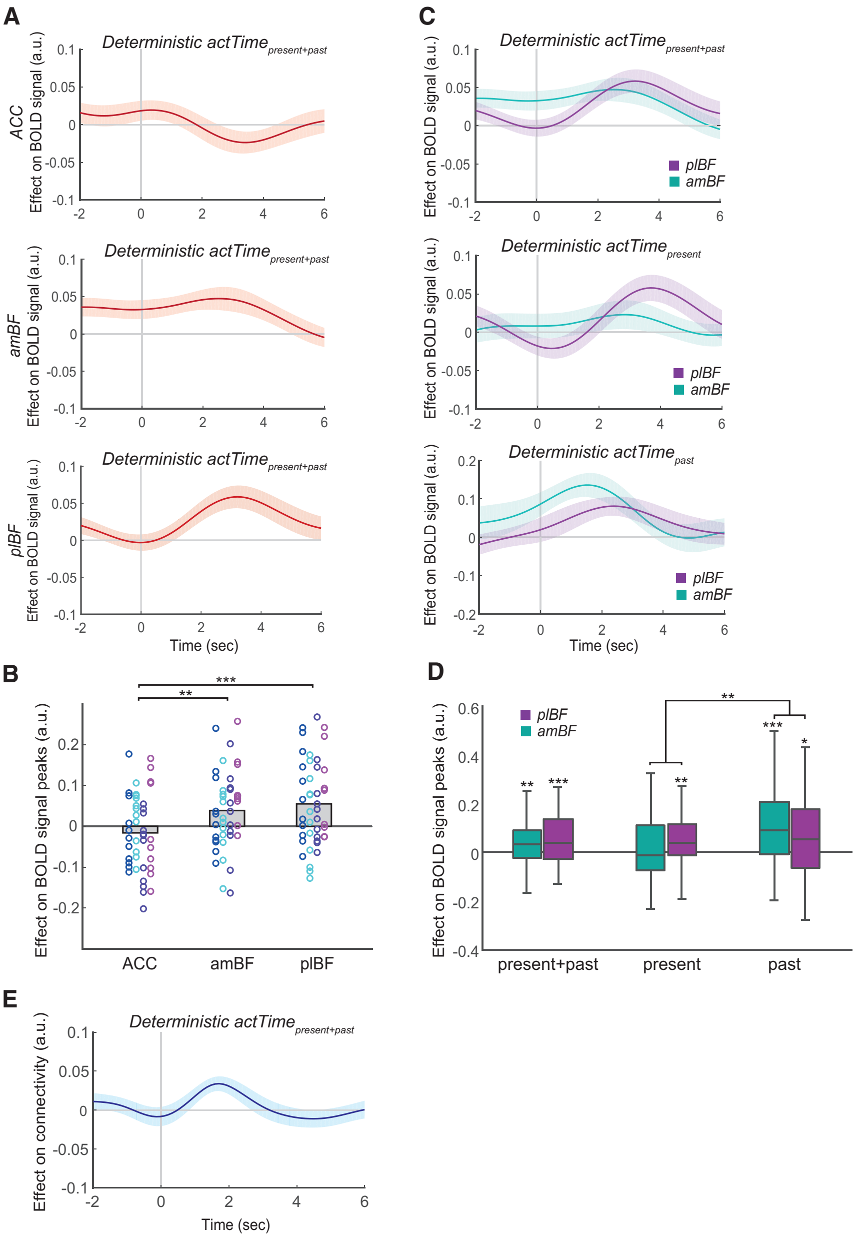

Figure 5. The BF Encodes the Deterministic Component of Time to Act

(A) ROI time course analysis of the ACC, amBF, and plBF, showing the relationship between BOLD activity and deterministic actTime estimated from present and past context. Format is as in Figure 4B.

(B) The relationship between deterministic actTime and BOLD signal was significantly stronger in the plBF and amBF compared with the ACC. Format is as in Figure 4C.

(C) ROI time course analysis of the amBF and plBF, showing the relationship between BOLD activity and deterministic actTime as estimated from present and past, present, and past contextual factors.

(D) The relationship between deterministic actTime, as estimated from past contextual factors, and BOLD signal was significantly stronger in the amBF compared with the plBF. In boxplots, the central line indicates the median, and the bottom and top edges of the box indicate the 25th and 75th percentiles, respectively. Whiskers extend to the most extreme data points not considered outliers.

(E) PPI analysis between the BOLD signal in the plBF and amBF, with deterministic actTime as the psychological factor. Trial-by-trial variation in the activity in the plBF was significantly related to trial-by-trial variation in the activity in the amBF as a function of deterministic actTime.

One-sample t tests and multilevel ANOVA followed by pairwise t test. *$p < 0.05$, **$p < 0.01$, ***$p < 0.001$. See also Figure S4.

with respect to response onset. ROIs were defined as spheres centered on the peak of the activations (Figure 4B; Figure S3 illustrates the time course of each contextual factor). We found no significant difference in activity between these three areas (multilevel ANOVA, $\chi^2(2) = 0.77$, $p = 0.68$) (Figure 4C). Given the delay in the hemodynamic response (BOLD signals take approximately 3 s to peak in the monkey; Chau et al., 2015), it is clear that the activity in all three areas begins in advance of the response.

Trial-by-trial variation in dot speed decorrelates actTime, measured in number of dots, from passage of time, measured in seconds ($r = 0.36 \pm 0.28$, across all sessions, where r is the correlation coefficient). However, to make sure that the relationship between BOLD and observed actTime is not simply explained by passage of time, we ran a new GLM (STAR Methods, GLM2.2) but this time also added time to act in seconds to the model as a covariate. The result of the new model was similar to that obtained previously (compare Figures 4B and 4C and Figures S2B and S2C).

BF Integrates Contextual Factors to Construct the Deterministic Component of Time to Act

Whole-brain analyses suggested that ACC, amBF, and plBF activity is correlated with observed actTime. We next asked whether the same areas integrated contextual factors to compute the deterministic component of actTime, as estimated by the Cox regression model. We added deterministic actTime to the previous time series GLM as the variable of interest and the observed actTime (measured in both number of dots and seconds) as covariates (trial-by-trial correlation between deterministic and observed actTime as measured in number of dots and seconds are $r = 0.24 \pm 0.23$ and $r = 0.11 \pm 0.27$ across sessions, respectively) (STAR Methods, GLM2.3; Figure 5A). We found that deterministic actTime explained BOLD activity in the amBF (one-sample t test; $t(44) = 2.78$, $p = 0.008$, $d = 0.41$, where d is the effect size) and plBF ($t(44) = 3.59$, $p < 0.001$, $d = 0.54$). We found no significant relationship between deterministic actTime and BOLD in the ACC ($t(44) = -1.15$, $p = 0.26$) (Figure 5B;

leave-one-out procedure for peak selection). Multilevel ANOVA showed a significant main effect of ROI ($\chi^2(2) = 14.65$, p = 0.0006); deterministic actTime was a better predictor of BOLD signal in the plBF compared with the ACC (Tukey's HSD; β = 0.07 ± 0.02, *Z* = 3.82, p = 0.0004) and in the amBF compared with the ACC (β = 0.05 ± 0.02, *Z* = 2.93, p = 0.0095). We found no significant difference between plBF and amBF (β = 0.02 ± 0.02, *Z* = 0.88, p = 0.65). This suggests that the BF is more strongly involved in integrating present and past contextual information to construct the deterministic component of actTime, compared with the ACC. A model-based whole-brain analysis confirmed the importance of the BF (Figure S4).

Next we asked whether present and past contextual factors contribute equally to encoding of deterministic actTime in the BF. Deterministic $\text{actTime}_{\text{present context}}$ and deterministic $\text{actTime}_{\text{past context}}$ were used in a time-series GLM (STAR Methods, GLM2.4), with the observed actTime (measured in both number of dots and seconds) as covariate (Figure 5C). BOLD activity in the plBF was related with trial-by-trial variation in deterministic $\text{actTime}_{\text{present context}}$ (one-sample t test; $t(44)$ = 3.16, p = 0.003, *d* = 0.47) and deterministic $\text{actTime}_{\text{past context}}$ ($t(44)$ = 2.47, p = 0.017, *d* = 0.37). However, BOLD activity in the amBF was only related with deterministic $\text{actTime}_{\text{past context}}$ ($t(44)$ = 4.04, p = 0.0002, *d* = 0.60), but its relationship with $\text{actTime}_{\text{present context}}$ was not significant ($t(44)$ = 0.85, p = 0.40; Figure 5D). Importantly, we found a significant interaction between deterministic actTime (present and past versus present versus past) and BF (amBF versus plBF) (multilevel ANOVA; $\chi^2(2) = 7.78$, p = 0.02); the relationship between deterministic actTime, when estimated from past as opposed to present context, and BOLD was significantly stronger in the amBF compared with the plBF (β = 0.1 ± 0.04, $t(220)$ = 2.64, p = 0.009) (Figure 5D), suggesting that the amBF mostly employs past contextual factors to construct actTime. Its estimate of $\text{actTime}_{\text{past context}}$ may then be passed to the plBF and integrated with $\text{actTime}_{\text{present context}}$ to estimate time to act. Thus, we should predict that functional connectivity between the plBF and amBF is moderated by deterministic actTime. A psychophysiological interaction (PPI) analysis (O'Reilly et al., 2012; STAR Methods, GLM2.5) confirmed that this was the case ($t(44)$ = 3.53, p = 0.001, *d = 0.52*) (Figure 5E). Although PPI cannot reveal the direction of influence, it is clear that the deterministic component of actTime is constructed within a circuit comprising both BF subdivisions. Finally, although timing differences in BOLD signals must be interpreted with care, it is noteworthy that, despite the proximity and similar nature of the two structures, amBF activity precedes plBF activity, and when the BOLD hemodynamic lag is considered, it is clear that amBF signaled $\text{actTime}_{\text{past context}}$ even during the previous ITI prior to trial onset (Figure 5C). In contrast, $\text{actTime}_{\text{present context}}$ is only encoded after trial onset, when the factors determining it are observable.

The Effect of Expected Reward on Time to Act Is Mediated by the ACC

Our findings so far suggest that the BF integrates present and past contextual factors to construct the deterministic component of actTime. However, although ACC's activity is correlated with actTime observed on any trial (Figure 4), its activity does not reflect what the actTime ought to be on any trial—the deterministic actTime—in a simple manner (Figure 5). We hypothesized that the ACC might mediate the effect of a specific element among contextual factors on actTime rather than a compound effect, as is the case with the BF. More specifically, given its known role in reward-guided decision-making (Kolling et al., 2016; Wittmann et al., 2016), we predicted that the observed relationship between reward magnitude and actTime (Figure 2C) might be mediated by the ACC. One method to test this hypothesis is mediation analysis (STAR Methods, Figure 6A). Mediation occurs when the direct effect of contextual factors on actTime (path c) can be explained by an indirect pathway through a brain area (path a × b). Note demonstrating mediation requires only that there be a significant indirect effect (path a × b) (Hayes, 2018; Zhao et al., 2010). This was indeed the case; the quadratic relationship between expected reward magnitude and actTime was mediated by an indirect effect through the ACC (bootstrapped p = 0.01). However, we did not find a mediation effect for the amBF (bootstrapped p = 0.91) or plBF (bootstrapped p = 0.24) (Figure 6B) or between other elements among contextual factors (dot speed, ITI, past reward; however, see the legend of Figure 6, past actTime) and actTime (all bootstrapped p > 0.2). This suggests that the ACC mediates the observed relationship between reward magnitude and actTime (Figure 2C). This reward-guided actTime could then be passed to the plBF to be used in construction of $\text{actTime}_{\text{present context}}$ along with $\text{actTime}_{\text{past context}}$ from the amBF. This would be consistent with the earlier timing of the ACC effect in relation to the plBF effect (Figure 4B), but it can be difficult to interpret timing differences in the BOLD signal, especially when they occur in spatially distant brain areas such as the ACC and plBF. A PPI analysis, however, was also consistent with this hypothesis; functional connectivity between the ACC and plBF ($t(44) = -2.07$, p = 0.044, *d* = 0.31) was moderated by quadratic reward magnitude (STAR Methods, GLM2.6). We did not find a moderation effect of reward magnitude on the functional connectivity between the ACC and amBF ($t(44)$ = 0.81, p = 0.42). This suggests that, by encoding the relationship between expected reward and actTime, the ACC contributes to construction of actTime mainly through connectivity with the plBF rather than the amBF (Wilcoxon signed-rank test; *Z* = 2.11, p = 0.035, *r* = 0.22) (Figure 6C). In summary, the ACC is modulated by reward magnitude (Figure S3A) and by the relationship between reward magnitude and action time (Figure 6), but unlike the BF (Figure 5), it does not integrate contextual factors from the present and past to determine how long animals should wait before responding at each trial.

Ultrasound Stimulation Selectively and Effectively Modulates Activity in Deep Brain Areas

It has been shown that 40 s sonification at 250 kHz reaches deep cortical areas such as the ACC and subcortical brain areas such as the amygdala and does so in a relatively focal manner, having less effect on adjacent, overlying brain areas (Folloni et al., 2019). We therefore first examined whether TUS could also be used to manipulate activity in an even deeper structure, the BF. To verify whether TUS effectively modulates BF activity, we first simulated the acoustic wave propagation and its thermal effect in a whole-head, finite-element model based on a high-resolution monkey

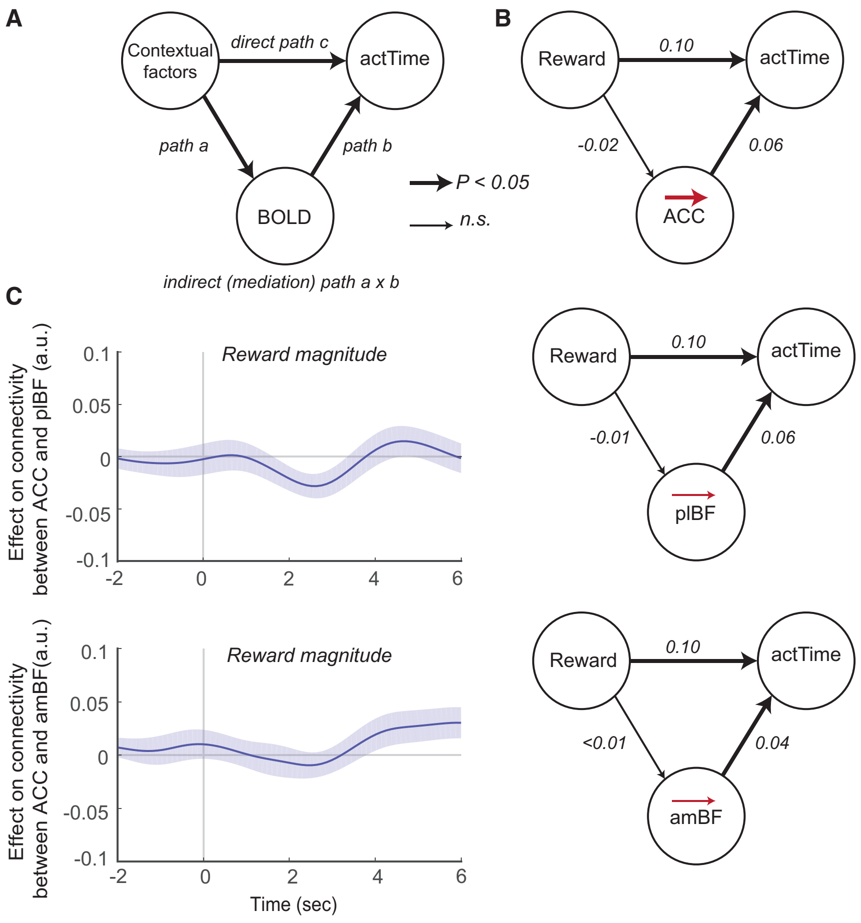

Figure 6. Mediation Analysis Can Explain the Relationship between Reward Magnitude and actTime

(A) Mediation occurs when the direct effect of contextual factors on actTime (path c) can be explained by an indirect pathway through a brain area (path a × b).

(B) The ACC mediated the quadratic relationship between reward magnitude and actTime. This was not the case for plBF or amBF or between other elements among contextual factors. However, after controlling for the effect of reward magnitude on the current trial, we found a small mediation effect of the ACC for the influence of past reward on actTime (bootstrapped p = 0.06). The numbers are the coefficients of each path. Significant paths are displayed with thick arrows.

(C) PPI analysis between the BOLD signal in the ACC and plBF (top panel) and amBF (bottom panel), with quadratic reward magnitude as the psychological factor. Trial-by-trial variation in the activity in the ACC was more strongly related with trial-by-trial variation in the activity in the plBF as a function of reward magnitude compared with the amBF (Wilcoxon signed-rank test; $Z = 2.11$, $p = 0.035$, $r = 0.22$).

computed tomography (CT) scan (Figure S5). The resulting "impact probability map" showed that the peak of the expected neuromodulatory effect was at the BF target (Figure 7A). We then performed offline rs-fMRI under anesthesia immediately after BF TUS in three animals (STAR Methods) and compared the data with rs-fMRI from three anesthetized control animals without TUS (Verhagen et al., 2019). Previous investigations of the neural effects of TUS have found that activity coupling between the targeted area and anatomically interconnected areas is altered (Folloni et al., 2019; Verhagen et al., 2019). The activity coupling of other brain areas, however, is unaffected. We might therefore expect altered coupling after BF TUS that is restricted to BF, but possibly extending to strongly connected regions such as the ACC. However, the BF projects broadly across many brain areas, and it has recently been reported that global rs-fMRI fluctuations across the whole brain are suppressed after BF perturbation (Turchi et al., 2018). Therefore, it is also possible that there will be widespread suppression of coupling elsewhere in the brain after BF TUS. We can test these predictions by quantifying the effect of TUS on brain activation by regressing the whole-brain connectivity profile of a seed area under the control condition against that observed after BF TUS. Importantly, we can repeat this regression analysis seeded for every point in the brain and report a map with scaling factors indicative of the TUS effect across the whole brain (Figure 7B). Indeed, we observed enhanced coupling in the BF target site and selectively connected regions, such as the ACC, but not elsewhere. Although the BF projects to many cortical areas, its connections with the ACC are notable because the ACC also projects to the BF (Ghashghaei and Barbas, 2001). This is, of course, also consistent with the evidence that we have already presented that the BF and ACC act in concert to influence actTime. In contrast, there was widespread suppression of coupling elsewhere in the brain after BF TUS compared with the control (Figure 7B). These effects of TUS were specific to stimulation of the BF and not observed after TUS of another region, as evidenced by seed-based correlation analyses following TUS over the supplementary motor area (SMA) (Verhagen et al., 2019; Figure S6).

Ultrasound Stimulation of the ACC and BF Modulates Time to Act

If the effect of contextual factors on actTime is mediated by the BF and ACC, then causal manipulation of these areas should modulate the relationship between contextual factors and actTime. We collected a new dataset outside of the scanner, but this time we used TUS to modulate ACC and BF activity immediately before animals performed the task (STAR Methods). Each animal underwent four stimulation conditions, including two conditions of interest and two control conditions (Figure 7C). Our two primary conditions were bilateral ACC TUS and bilateral BF TUS. We also chose two control conditions: passive bilateral sham control with the transducer positioned on the skull but without sonication and one active control with TUS targeted at the bilateral parietal operculum (POp), a region

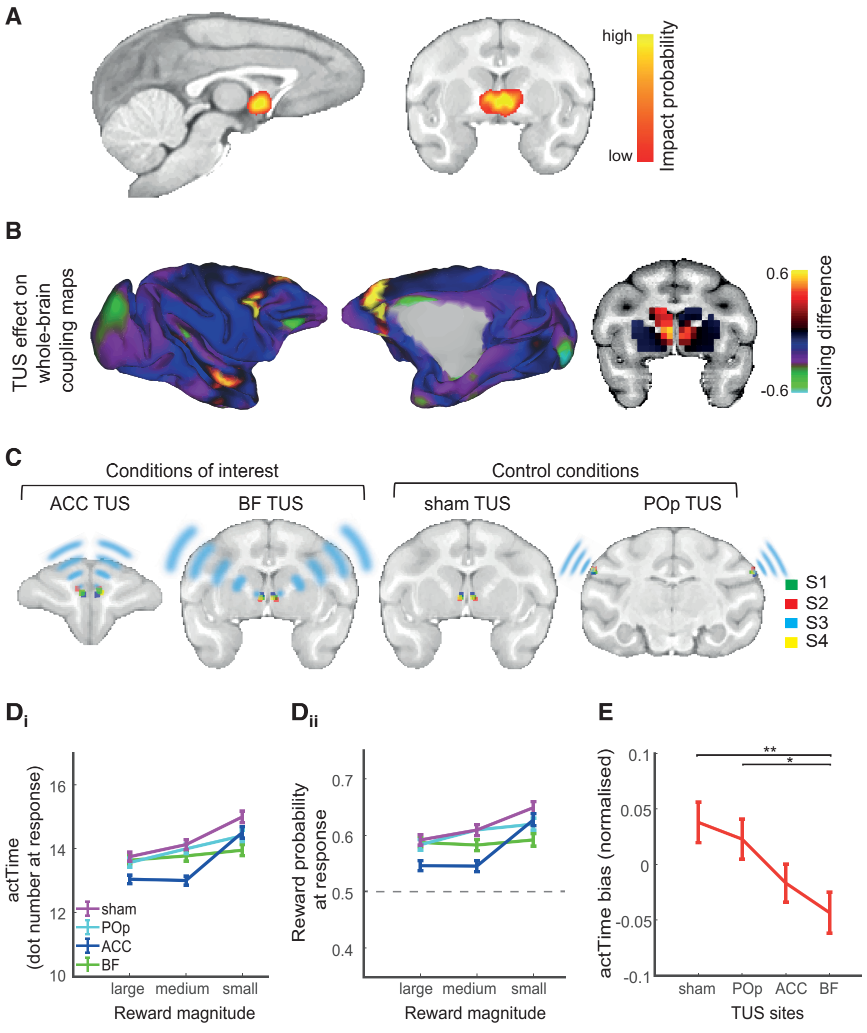

Figure 7. Ultrasound Stimulation of the ACC and BF Modulates Time to Act

(A) Probability map of the combined neural impact of sonications targeted at the BF. This is calculated as the average stimulation intensity (I_{sppa}) map across the two consecutive stimulations delivered over the left and right hemisphere, respectively (Figure S5). The combined impact probability map is overlaid on a standard F99 brain. The low impact probability level corresponds to 10 W/cm^2, in correspondence with previous work (Verhagen et al., 2019) and Figure S5. As the color changes from red to bright yellow, the probability of neuromodulation from bilateral BF TUS increases.

(B) The result of a regression analysis comparing, for each point in the brain, its whole-brain coupling map ("connectome") in the no-stimulation state against its coupling map observed after TUS targeted at the BF. The hot colors indicate points in the brain with enhanced whole-brain coupling strength after BF TUS compared with no TUS, whereas the cool colors indicate reduced whole-brain coupling strength after BF TUS compared with no TUS. Compared with no TUS, BF TUS showed a clear enhancement in activity coupling within the BF and between the BF and ACC and superior temporal area (Ts2).

(C) Sites where TUS was applied for each animal (S1, S2, S3, and S4) to assess its effect on actTime. The TUS transducer was set at a resonance frequency of 250 kHz and concentrated ultrasound in a cigar-shaped focal spot in the ACC, BF, and POp. For the sham control, the transducer was positioned on the skull but without sonication.

(D) Animals acted more quickly after ACC than after BF, POp, or sham TUS when offered medium or large compared with small rewards. actTime is indexed as the number of dots at response (D_i) and the corresponding reward probability (D_{ii}).

(E) BF TUS reduced the bias between observed and deterministic actTime compared with POp and sham TUS.

Error bars show SEM across observations. Multilevel ANOVA followed by pairwise t test. *$p < 0.05$, **$p < 0.01$, ***$p < 0.001$.

See also Figures S5–S7.

distinct from and anterior to intraparietal areas linked to sensorimotor decision-making (Shadlen and Kiani, 2013), in which activity was unmodulated by our contrast of interest or any other task event. Each condition was repeated five times on separate days in a pseudo-randomized order for each animal (20 sessions per animal).

Based on the findings from the mediation analysis, we first predicted that ACC TUS should modulate the relationship between reward magnitude and actTime compared with control conditions. Multilevel ANOVA (GLM3.1) showed a significant interaction between TUS and reward magnitude ($\chi^2(6) = 26.89$, $p = 0.0002$). Planned contrasts (STAR Methods) revealed that animals acted quicker after ACC TUS than after BF, control POp, or sham TUS but only when offered a medium reward (compared with a small one) (ACC versus BF, $\beta = -1.35 \pm 0.31$, $t(13{,}068) = -4.34$, $p < 0.001$; ACC versus POp, $\beta = -1.13 \pm 0.31$, $t(13{,}068) = -3.62$, $p < 0.001$; ACC versus sham, $\beta = -0.66 \pm 0.31$, $t(13{,}068) = -2.11$, $p = 0.035$) or a large reward (compared with a small one) (ACC versus BF, $\beta = -1.16 \pm 0.30$, $t(13{,}068) = -3.78$, $p < 0.001$; ACC versus POp, $\beta = -0.65 \pm 0.30$, $t(13068) = -2.13$, $p = 0.033$; ACC versus sham did not reach significance) (Figure 7D_i; see Figure S7 for individual animal data). This pattern of results confirms the interpretation of the fMRI results and suggests that the ACC causally mediates the relationship between reward magnitude and actTime. Interestingly, the effect was observed for large and medium levels of reward. Although the higher reward prospects on such trials make them enticing, it is important to wait before responding to increase the likelihood of success. Because ACC TUS compromised the ability to delay responding on just such trials, it led to a reduced likelihood of receipt of large rewards (Tukey's HSD; ACC versus sham, $\beta = -0.05 \pm 0.01$, $Z = -4.04$, $p < 0.001$; ACC versus POp, $\beta = -0.04 \pm 0.01$, $Z = -2.87$, $p = 0.022$; ACC versus BF, $\beta = -0.04 \pm 0.01$, $Z = -3.21$, $p = 0.007$; BF versus sham, $Z = -0.80$, $p = 0.85$; BF versus POp, $Z = 0.004$, $p = 0.99$) (Figure 7D_{ii}).

Previous studies showed that the TUS effect starts to diminish after approximately 1 h but is still evident up to 2 h after the end of stimulation (Folloni et al., 2019; Verhagen et al., 2019). In our experiment, the task was performed within 40 min of stimulation, well within the 1-h peak effect of TUS. Therefore, we do not expect to see a diminishing effect of stimulation on the behavior recorded during the task period. Nevertheless, we repeated the same analyses as before, but this time added ''time passed since beginning of the session'' as a covariate. This did not influence the results. Finally, we ran a control analysis by including all present and past contextual factors to check whether modulation of ACC activity could influence the relationship between other contextual factors (dot speed, ITI, past reward outcome, and past actTime) and actTime. We found no interaction between contextual factors and stimulation conditions (all $p > 0.16$) other than with reward magnitude ($\chi^2(6) = 37.30$, $p < 0.001$).

Having shown that TUS could be used to target the BF, we next investigated its behavioral effect. We hypothesized, based on our previous findings, that causal manipulation of the BF might have a stronger effect on the relationship between deterministic and observed actTime on a trial-by-trial basis compared with ACC, control POp, or sham stimulation. To quantify this relationship, we derived a measure of trial-by-trial actTime bias by subtracting each trial's deterministic actTime from the observed actTime and took the absolute value under each stimulation condition (STAR Methods, GLM3.2). Multilevel ANOVA showed a significant main effect of stimulation condition on actTime bias ($\chi^2(3) = 12.32$, $p = 0.006$); however, this effect was not observed across all animals (Figure S7). Planned contrasts (STAR Methods) showed that BF TUS reduced the bias between observed and deterministic actTime compared with both control POp ($\beta = 0.28 \pm 0.11$, $t(12,430) = 2.45$, $p = 0.014$) and sham ($\beta = 0.36 \pm 0.11$, $t(12,430) = 3.18$, $p = 0.001$) TUS (Figure 7E; Figure S7). The differences between the effects of BF and ACC TUS on this measure, when compared directly, were not significant (although, notably, post hoc tests showed no significant difference between ACC and POp or sham TUS, either). This overall pattern of behavior change after TUS is consistent with the recording data that suggested that, although the BF had the key role in the encoding of actTime, the BF and ACC are strongly connected and interact (Figures 6 and 7B; Figure S6).

DISCUSSION

Although a number of studies have considered the important question of how an action is initiated, here we identified factors that influence when (Figures 2A–2E) and even whether (Figure 2F) an action should be initiated. These factors relate to both the current context, signaled to the animal by cues in the environment, and the recent past context, such as recent rewards and the timing of recent previous decisions (Figure 3). Even though a large fraction of variance in animals' behavior remains unexplained, by careful and controlled manipulation of identifiable features of the environment, we managed to explain a considerable proportion of variance in their decision time to act (Figure 3E).

Few theoretical accounts exist to explain why actions might be made at one time rather than another, and none is quite adequate to explain all of the observations made in the current study. Potentially marginal value theorem (MVT), which predicts many aspects of decision-making (Pearson et al., 2014), might be used to predict when actions will be made. In the fMRI experiment, macaques responded more deliberately and slowly when there was a possibility of large reward as opposed to a medium reward (Figure S1). Careful long actTimes ensure that opportunities to obtain a large reward are not wasted before transitioning to the next trial when the possibility of another large reward is lower and equal to the possibility of a small or medium reward. However, many aspects of the current findings are not explained easily by MVT because it predicts that animals should respond faster when their intake rate diminishes to the average intake rate of the environment (Charnov, 1976; Krebs, 1987). In contrast, we found that on small reward trials actions were made more slowly rather than more quickly. These aspects of deciding when to act were consistent with an alternative notion that vigor and speed of responding increase as the average reward rate increases; actions are made quickly so that reward opportunities are not lost, whereas slow actions entail an opportunity cost (Niv et al., 2007). Finally, in line with observations made in rodents (Murakami et al., 2014, 2017), we observed that an additional important predictor of the timing of the next action is the timing of recent past actions.

We used fMRI to look for brain activity that is parametrically related to the factors that change the likelihood of action. Note that we are not looking for brain areas that initiate the action but, rather, those that encode the current and recent past factors that influence the right time to make the action. We identified two areas (Figure 4): the ACC and BF. However, the influence of present and past context on when an action will emerge was more strongly encoded by the BF compared with the ACC; BOLD activity in the BF could be explained by trial-by-trial variation in deterministic actTime, which is the predicted actTime given present and past contextual factors. Moreover, compared with control POp TUS and sham TUS, manipulation of the BF with TUS significantly altered the closeness of the relationship between deterministic actTime and the actually observed actTime (Figure 7E). In contrast, ACC TUS did not change this relationship in comparison with control POp TUS and sham TUS.

BF activity extended across both the amBF (approximately the medial septum/diagonal band of Broca) and plBF (approximately the *nucleus basalis* of Meynert) (Figure 4). Although plBF activity was significantly correlated with deterministic actTime predicted from both present and past contextual factors, amBF activity was only correlated with deterministic actTime predicted from past contextual factors. The animal's internal state (shaped by past trials) may be passed from the amBF to the plBF and integrated with present contextual factors to inform time to act in the plBF. Some aspects of the influence of present contextual factors on construction of deterministic actTime involve the ACC; the ACC mediated the effect of reward magnitude on actTime. PPI analyses were consistent with a circuit comprising both the BF and ACC in which the component parts of the BF, the amBF and plBF, were connected as a function of deterministic actTime, whereas current reward magnitude influenced the functional connectivity between the ACC and plBF (Figure 8).

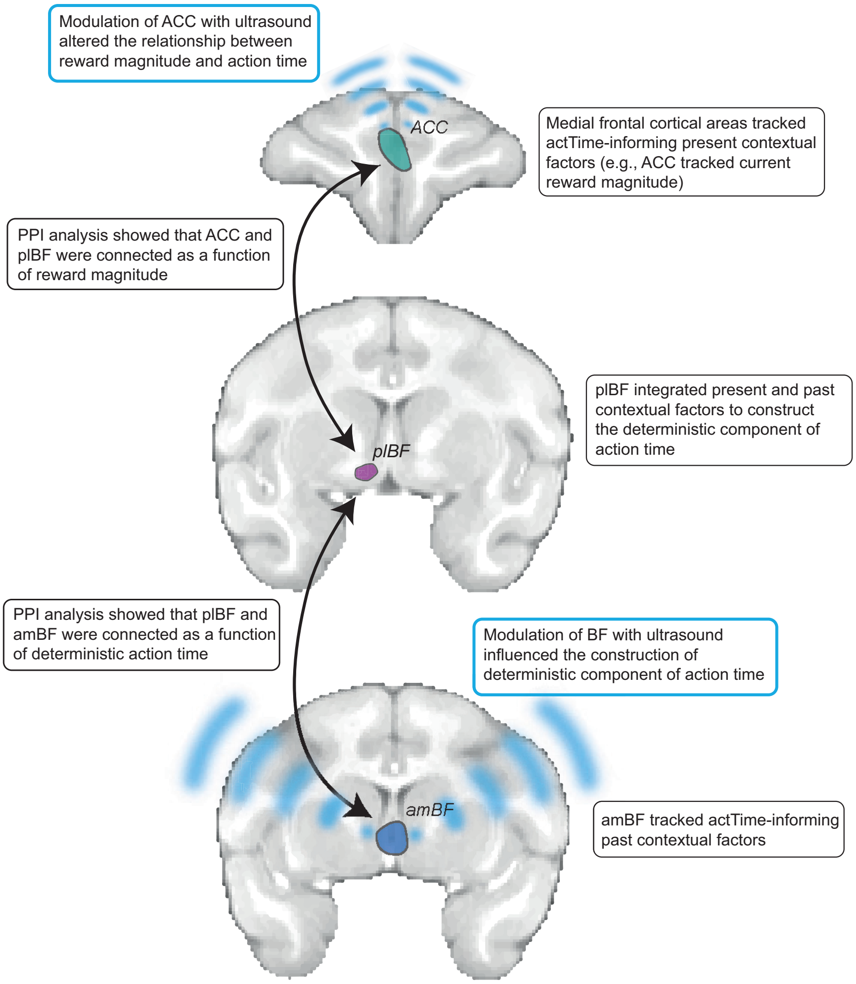

Figure 8. A Schematic View of the Main Findings

A BF-cingulate circuit in macaques decides it is time to act by integrating identifiable features of both the current context and the recent past context.

Within the cortex, voluntary action has been especially linked to medial frontal areas such as the ACC (Heilbronner and Hayden, 2016; Thaler et al., 1995) or closely adjacent areas such as the SMA and pre-SMA (Lara et al., 2018). Although investigations of such areas have made clear that the medial frontal cortex is important when action is initiated voluntarily, the precise role has been less clear. The present results demonstrate that it is the influence of the prospect of reward on the decision about when to act that is mediated by the ACC. This is consistent with the fact that the ACC carries multiple value signals (Kolling et al., 2016; Meder et al., 2017) but, at the same time, has also been associated with determining the balance between persistence with a given manner of responding or changing to an alternative mode (Ebitz et al., 2018; Karlsson et al., 2012; Parvizi et al., 2013; Stoll et al., 2016b; Wittmann et al., 2016), when to leave a depleting patch in a foraging task (Hayden et al., 2011), and signaling initiation of an action plan by integrating evidence toward a decision bound (Hunt et al., 2018). We did not find a clear mediation effect for the influence of past reward on action time (Figure 6). This was unexpected, given previous studies showing the role of the ACC in encoding of reward history (Kennerley et al., 2006; Seo and Lee, 2007). It may reflect the limited influence of this one factor on actTime, given the many other factors that also affected actTime. Nevertheless, we found a direct effect of past trial reward outcome on the ACC, irrespective of its effect on actTime (Figure S3D), and there was a suggestion, from a marginally significant result, of a degree of mediation of the influence of the past reward on actTime (see the legend of Figure 6).

The prospect of reward may have a complex effect on the determination of when to act. On one hand, when a reward is available, it may be adaptive to increase the response rate (Niv et al., 2007). On the other hand, if there is a particularly large reward available right now on the current trial (e.g., high-reward trials in the current task) and there is only a small chance that the same level of reward will be available on the next trial (there is only a 0.33 probability that the next trial will be a high-reward trial), then care should be taken in how the response is made so that the current high-reward opportunity is not wasted. It was notable that ACC disruption by TUS particularly led to faster responses on medium- and high-reward trials (Figure 7D_i), which meant that animals were less likely to actually receive the medium and high rewards (Figure 7D_{ii}). In some cases (e.g., Figure S1), animals produced carefully controlled, slow responses on high-reward trials. Danielmeier et al., 2015 have also inferred, from recordings of ACC activity and cholinergic pharmacological investigation, that the ACC might mediate controlled response strategies via the BF. This is consistent with the negative direction of the PPI effect (Figure 6C), consistent with an inhibitory influence between the ACC and plBF when carefully controlled responding was needed on higher reward expectation trials.

Although many aspects of TUS, such as its ability to stimulate deep in the brain while leaving overlying areas unaffected, compare favorably with other minimally invasive stimulation techniques, there are still limits to its precision (in the present study, BF TUS spreads to both active sub-regions). However, although the precision of TUS is likely to improve to allow more specific targeting in the future, it will also be important to relate the findings to activity patterns recorded in specific neuron populations. BF is a major neuromodulatory hub. It is the major source of cholinergic projection neurons to the cortex (Mesulam et al., 1983). Although BF and acetylcholine has been linked to affect, attention, motivation, and memory (Danielmeier et al., 2015; Marshall et al., 2016; Záborszky et al., 2018), our demonstration that BF mediates the

influence of past and present context on the emergence of a decision of when to act might seem surprising, especially given that the nigrostriatal dopaminergic pathway has been linked to self-paced action initiation (Howe and Dombeck, 2016; da Silva et al., 2018). We cannot completely rule out the influence of BF TUS on adjacent basal ganglion regions. However, a recent study shows that dopamine and acetylcholine may play independent and complementary roles in initiation of self-paced actions (Howe et al., 2019), and other studies indicate that BF activity is linked to response initiation and suppression (Avila and Lin, 2014; Mayse et al., 2015). Moreover, neurons in the medial BF of monkeys combine various contextual factors, such as reward size and uncertainty (Monosov et al., 2015). Some of these neurons have ramping activity that anticipates the timing of external events (Zhang et al., 2019). Here we suggest a new function for a BF-cingulate circuit in combining contextual factors with internal state to guide decisions about when to act or, equally, when not to act. This finding could be of potential clinical importance, given the involvement of the BF cholinergic system in Parkinson's disease (Ballinger et al., 2016). Future studies should assess the possible distinctive role of cholinergic and noncholinergic BF neurons in decisions about when to act.

Consideration of previous investigations of nigrostriatal activity in tandem with the current results suggests the hypothesis that the BF integrates past and present contextual information that will influence the decision about when an action should be made and communicates this information to nigrostriatal circuits via direct or indirect pathways known to exist between them (Hikosaka, 2010). It is in the nigrostriatal circuit, or in one of the interconnecting linking regions such as the habenula (Matsumoto and Hikosaka, 2007), that action initiation per se begins.

STAR★METHODS

Detailed methods are provided in the online version of this paper and include the following:

- KEY RESOURCES TABLE
- LEAD CONTACT AND MATERIALS AVAILABILITY
- EXPERIMENTAL MODEL AND SUBJECT DETAILS
- METHOD DETAILS
  - Behavioral Training
  - Experimental task
  - Cox regression model
  - Imaging data acquisition
  - fMRI data preprocessing
  - Transcranial Ultrasound Stimulation (TUS)
  - Acoustic and thermal modeling
  - Resting-state imaging data acquisition, pre-processing, and analysis
- QUANTIFICATION AND STATISTICAL ANALYSIS
  - Behavioral analysis
  - fMRI data analysis
  - ROI analysis
  - Leave one out on time-series group peak signal
  - Mediation analysis
  - TUS behavioral data analysis
- DATA AND CODE AVAILABILITY

SUPPLEMENTAL INFORMATION

Supplemental Information can be found online at https://doi.org/10.1016/j.neuron.2019.10.030.

ACKNOWLEDGMENTS

This work was funded by the Wellcome Trust (203139/Z/16/Z, WT100973AIA, WT101092MA, 105238/Z/14/Z, and 105651/Z/14/Z), the Medical Research Council (MR/P024955/1 and MRC G0902373), the Bettencourt Schueller Foundation, and Agence Nationale de la Recherche under the Future Investments program (ANR-10-EQPX-15). We are very grateful to Dr. Masayoshi Murakami and Dr. Zach Mainen for sharing the analysis code and to Oxford University Biomedical Services staff for ensuring the welfare of animals.

AUTHOR CONTRIBUTIONS

N.K. and M.F.S.R. designed the experiments. N.K. and A.B. trained the animals. N.K. collected the data. N.K., L.V., and M.F.S.R. analyzed the data. N.K., D.F., and J.S. performed TUS. D.A., and J.-F.A. performed the acoustic and thermal simulations. N.K. and M.F.S.R. wrote the manuscript. All authors read and approved the final version of the paper.

DECLARATION OF INTERESTS

The authors declare no competing interests.

Received: July 23, 2019
Revised: October 2, 2019
Accepted: October 22, 2019
Published: December 5, 2019

SUPPORTING CITATIONS

The following references appear in the Supplemental Information: Andrews and Heathcote (2001); Balota et al. (2008); Cavanagh et al. (2016); Hunt et al. (2015); Kennerley et al. (2009); Lewandowsky and Farrell (2011); Luce (1991).

REFERENCES

Airan, R.D., and Butts Pauly, K. (2018). Hearing out Ultrasound Neuromodulation. Neuron *98*, 875–877.

Andrews, S., and Heathcote, A. (2001). Distinguishing common and task-specific processes in word identification: a matter of some moment? J. Exp. Psychol. Learn. Mem. Cogn. *27*, 514–544.

Aubry, J.F., Tanter, M., Pernot, M., Thomas, J.L., and Fink, M. (2003). Experimental demonstration of noninvasive transskull adaptive focusing based on prior computed tomography scans. J. Acoust. Soc. Am. *113*, 84–93.

Avila, I., and Lin, S.C. (2014). Motivational salience signal in the basal forebrain is coupled with faster and more precise decision speed. PLoS Biol. *12*, e1001811.

Ballinger, E.C., Ananth, M., Talmage, D.A., and Role, L.W. (2016). Basal Forebrain Cholinergic Circuits and Signaling in Cognition and Cognitive Decline. Neuron *91*, 1199–1218.

Balota, D.A., Yap, M.J., Cortese, M.J., and Watson, J.M. (2008). Beyond Mean Response Latency: Response Time Distributional Analyses of Semantic Priming. J. Mem. Lang. *59*, 495–523.

Bates, D., Maechler, M., Bolker, B., Walker, S., Christensen, R.H.B., Singmann, H., Dai, B., Scheipl, F., Grothendieck, G., Green, P., et al. (2018). lme4: Linear Mixed-Effects Models using "Eigen" and S4. Journal of Statistical Software *67*.

Cavanagh, S.E., Wallis, J.D., Kennerley, S.W., and Hunt, L.T. (2016). Autocorrelation structure at rest predicts value correlates of single neurons during reward-guided choice. eLife *5*, e18937.

Charnov, E.L. (1976). Optimal foraging, the marginal value theorem. Theor. Popul. Biol. *9*, 129–136.

Chau, B.K.H., Sallet, J., Papageorgiou, G.K., Noonan, M.P., Bell, A.H., Walton, M.E., and Rushworth, M.F.S. (2015). Contrasting Roles for Orbitofrontal Cortex and Amygdala in Credit Assignment and Learning in Macaques. Neuron *87*, 1106–1118.

Constans, C., Deffieux, T., Pouget, P., Tanter, M., and Aubry, J.F. (2017). A 200-1380-kHz Quadrifrequency Focused Ultrasound Transducer for Neurostimulation in Rodents and Primates: Transcranial In Vitro Calibration and Numerical Study of the Influence of Skull Cavity. IEEE Trans. Ultrason. Ferroelectr. Freq. Control *64*, 717–724.

Constans, C., Mateo, P., Tanter, M., and Aubry, J.F. (2018). Potential impact of thermal effects during ultrasonic neurostimulation: retrospective numerical estimation of temperature elevation in seven rodent setups. Phys. Med. Biol. *63*, 025003.

Cox, B.T., Kara, S., Arridge, S.R., and Beard, P.C. (2007). k-space propagation models for acoustically heterogeneous media: application to biomedical photoacoustics. J. Acoust. Soc. Am. *121*, 3453–3464.

da Silva, J.A., Tecuapetla, F., Paixão, V., and Costa, R.M. (2018). Dopamine neuron activity before action initiation gates and invigorates future movements. Nature *554*, 244–248.

Danielmeier, C., Allen, E.A., Jocham, G., Onur, O.A., Eichele, T., and Ullsperger, M. (2015). Acetylcholine mediates behavioral and neural post-error control. Curr. Biol. *25*, 1461–1468.

Duck, F.A. (2012). Physical Properties of Tissue: A Comprehensive Reference Book (Institute of Physics and Engineering in Medicine).

Ebitz, R.B., Albarran, E., and Moore, T. (2018). Exploration Disrupts Choice-Predictive Signals and Alters Dynamics in Prefrontal Cortex. Neuron *97*, 450–461.e9.

Folloni, D., Verhagen, L., Mars, R.B., Fouragnan, E., Constans, C., Aubry, J.F., Rushworth, M.F.S., and Sallet, J. (2019). Manipulation of Subcortical and Deep Cortical Activity in the Primate Brain Using Transcranial Focused Ultrasound Stimulation. Neuron *101*, 1109–1116.e5.

Fouragnan, E.F., Chau, B.K.H., Folloni, D., Kolling, N., Verhagen, L., Klein-Flügge, M., Tankelevitch, L., Papageorgiou, G.K., Aubry, J.F., Sallet, J., and Rushworth, M.F.S. (2019). The macaque anterior cingulate cortex translates counterfactual choice value into actual behavioral change. Nat. Neurosci. *22*, 797–808.

Fritz, H.J., Ray, N., Dyrba, M., Sorg, C., Teipel, S., and Grothe, M.J. (2019). The corticotopic organization of the human basal forebrain as revealed by regionally selective functional connectivity profiles. Hum. Brain Mapp. *40*, 868–878.

Ghashghaei, H.T., and Barbas, H. (2001). Neural interaction between the basal forebrain and functionally distinct prefrontal cortices in the rhesus monkey. Neuroscience *103*, 593–614.

Glasser, M.F., Sotiropoulos, S.N., Wilson, J.A., Coalson, T.S., Fischl, B., Andersson, J.L., Xu, J., Jbabdi, S., Webster, M., Polimeni, J.R., et al.; WU-Minn HCP Consortium (2013). The minimal preprocessing pipelines for the Human Connectome Project. Neuroimage *80*, 105–124.

Goss, S.A., Frizzell, L.A., and Dunn, F. (1979). Ultrasonic absorption and attenuation in mammalian tissues. Ultrasound Med. Biol. *5*, 181–186.

Hayden, B.Y., Pearson, J.M., and Platt, M.L. (2011). Neuronal basis of sequential foraging decisions in a patchy environment. Nat. Neurosci. *14*, 933–939.

Hayes, A.F. (2018). Introduction to mediation, moderation, and conditional process analysis: a regression-based approach (The Guilford Press).

Heilbronner, S.R., and Hayden, B.Y. (2016). Dorsal Anterior Cingulate Cortex: A Bottom-Up View. Annu. Rev. Neurosci. *39*, 149–170.

Hikosaka, O. (2010). The habenula: from stress evasion to value-based decision-making. Nat. Rev. Neurosci. *11*, 503–513.

Howe, M.W., and Dombeck, D.A. (2016). Rapid signalling in distinct dopaminergic axons during locomotion and reward. Nature *535*, 505–510.

Howe, M., Ridouh, I., Allegra Mascaro, A.L., Larios, A., Azcorra, M., and Dombeck, D.A. (2019). Coordination of rapid cholinergic and dopaminergic signaling in striatum during spontaneous movement. eLife *8*, e44903.

Hunt, L.T., Behrens, T.E.J., Hosokawa, T., Wallis, J.D., and Kennerley, S.W. (2015). Capturing the temporal evolution of choice across prefrontal cortex. eLife *4*, 4.

Hunt, L.T., Malalasekera, W.M.N., de Berker, A.O., Miranda, B., Farmer, S.F., Behrens, T.E.J., and Kennerley, S.W. (2018). Triple dissociation of attention and decision computations across prefrontal cortex. Nat. Neurosci. *21*, 1471–1481.

Jenkinson, M., Beckmann, C.F., Behrens, T.E.J., Woolrich, M.W., and Smith, S.M. (2012). FSL. Neuroimage *62*, 782–790.

Kagan, I., Iyer, A., Lindner, A., and Andersen, R.A. (2010). Space representation for eye movements is more contralateral in monkeys than in humans. Proc. Natl. Acad. Sci. USA *107*, 7933–7938.

Karlsson, M.P., Tervo, D.G.R., and Karpova, A.Y. (2012). Network resets in medial prefrontal cortex mark the onset of behavioral uncertainty. Science *338*, 135–139.

Kennerley, S.W., Walton, M.E., Behrens, T.E.J., Buckley, M.J., and Rushworth, M.F.S. (2006). Optimal decision making and the anterior cingulate cortex. Nat. Neurosci. *9*, 940–947.

Kennerley, S.W., Dahmubed, A.F., Lara, A.H., and Wallis, J.D. (2009). Neurons in the frontal lobe encode the value of multiple decision variables. J. Cogn. Neurosci. *21*, 1162–1178.

Kolling, N., Wittmann, M.K., Behrens, T.E.J., Boorman, E.D., Mars, R.B., and Rushworth, M.F.S. (2016). Value, search, persistence and model updating in anterior cingulate cortex. Nat. Neurosci. *19*, 1280–1285.

Kolster, H., Mandeville, J.B., Arsenault, J.T., Ekstrom, L.B., Wald, L.L., and Vanduffel, W. (2009). Visual field map clusters in macaque extrastriate visual cortex. J. Neurosci. *29*, 7031–7039.

Kolster, H., Janssens, T., Orban, G.A., and Vanduffel, W. (2014). The retinotopic organization of macaque occipitotemporal cortex anterior to V4 and caudoventral to the middle temporal (MT) cluster. J. Neurosci. *34*, 10168–10191.

Krebs, D.W.S.R. (1987). Foraging Theory by David W. Stephens (Princeton University Press).

Lara, A.H., Cunningham, J.P., and Churchland, M.M. (2018). Different population dynamics in the supplementary motor area and motor cortex during reaching. Nat. Commun. *9*, 2754.

Lewandowsky, S., and Farrell, S. (2011). Computational Modeling in Cognition: Principles and Practice (SAGE Publications, Inc).

Luce, R.D. (1991). Response Times: Their Role in Inferring Elementary Mental Organization (Oxford University Press).

Manohar, S.G., Chong, T.T.J., Apps, M.A.J., Batla, A., Stamelou, M., Jarman, P.R., Bhatia, K.P., and Husain, M. (2015). Reward Pays the Cost of Noise Reduction in Motor and Cognitive Control. Curr. Biol. *25*, 1707–1716.

Markello, R.D., Spreng, R.N., Luh, W.M., Anderson, A.K., and De Rosa, E. (2018). Segregation of the human basal forebrain using resting state functional MRI. Neuroimage *173*, 287–297.

Mars, R.B., Sallet, J., Neubert, F.X., and Rushworth, M.F.S. (2013). Connectivity profiles reveal the relationship between brain areas for social cognition in human and monkey temporoparietal cortex. Proc. Natl. Acad. Sci. USA *110*, 10806–10811.

Marshall, L., Mathys, C., Ruge, D., de Berker, A.O., Dayan, P., Stephan, K.E., and Bestmann, S. (2016). Pharmacological Fingerprints of Contextual Uncertainty. PLoS Biol. *14*, e1002575.

Matsumoto, M., and Hikosaka, O. (2007). Lateral habenula as a source of negative reward signals in dopamine neurons. Nature *447*, 1111–1115.

Mayse, J.D., Nelson, G.M., Avila, I., Gallagher, M., and Lin, S.C. (2015). Basal forebrain neuronal inhibition enables rapid behavioral stopping. Nat. Neurosci. *18*, 1501–1508.

Meder, D., Kolling, N., Verhagen, L., Wittmann, M.K., Scholl, J., Madsen, K.H., Hulme, O.J., Behrens, T.E.J., and Rushworth, M.F.S. (2017). Simultaneous representation of a spectrum of dynamically changing value estimates during decision making. Nat. Commun. *8*, 1942.

Mesulam, M.M., Mufson, E.J., Levey, A.I., and Wainer, B.H. (1983). Cholinergic innervation of cortex by the basal forebrain: cytochemistry and cortical connections of the septal area, diagonal band nuclei, nucleus basalis (substantia innominata), and hypothalamus in the rhesus monkey. J. Comp. Neurol. *214*, 170–197.

Mohammadjavadi, M., Ye, P.P., Xia, A., Brown, J., Popelka, G., and Pauly, K.B. (2019). Elimination of peripheral auditory pathway activation does not affect motor responses from ultrasound neuromodulation. Brain Stimul. *12*, 901–910.

Monosov, I.E., Leopold, D.A., and Hikosaka, O. (2015). Neurons in the Primate Medial Basal Forebrain Signal Combined Information about Reward Uncertainty, Value, and Punishment Anticipation. J. Neurosci. *35*, 7443–7459.

Murakami, M., Vicente, M.I., Costa, G.M., and Mainen, Z.F. (2014). Neural antecedents of self-initiated actions in secondary motor cortex. Nat. Neurosci. *17*, 1574–1582.

Murakami, M., Shteingart, H., Loewenstein, Y., and Mainen, Z.F. (2017). Distinct Sources of Deterministic and Stochastic Components of Action Timing Decisions in Rodent Frontal Cortex. Neuron *94*, 908–919.e7.

Nakahara, K., Hayashi, T., Konishi, S., and Miyashita, Y. (2002). Functional MRI of macaque monkeys performing a cognitive set-shifting task. Science *295*, 1532–1536.

Neubert, F.X., Mars, R.B., Sallet, J., and Rushworth, M.F.S. (2015). Connectivity reveals relationship of brain areas for reward-guided learning and decision making in human and monkey frontal cortex. Proc. Natl. Acad. Sci. USA *112*, E2695–E2704.

Niv, Y., Daw, N.D., Joel, D., and Dayan, P. (2007). Tonic dopamine: opportunity costs and the control of response vigor. Psychopharmacology (Berl.) *191*, 507–520.

O'Reilly, J.X., Woolrich, M.W., Behrens, T.E.J., Smith, S.M., and Johansen-Berg, H. (2012). Tools of the trade: psychophysiological interactions and functional connectivity. Soc. Cogn. Affect. Neurosci. *7*, 604–609.

Papageorgiou, G.K., Sallet, J., Wittmann, M.K., Chau, B.K.H., Schüffelgen, U., Buckley, M.J., and Rushworth, M.F.S. (2017). Inverted activity patterns in ventromedial prefrontal cortex during value-guided decision-making in a less-is-more task. Nat. Commun. *8*, 1886.

Parvizi, J., Rangarajan, V., Shirer, W.R., Desai, N., and Greicius, M.D. (2013). The will to persevere induced by electrical stimulation of the human cingulate gyrus. Neuron *80*, 1359–1367.

Pearson, J.M., Watson, K.K., and Platt, M.L. (2014). Decision making: the neuroethological turn. Neuron *82*, 950–965.

Pennes, H.H. (1948). Analysis of tissue and arterial blood temperatures in the resting human forearm. J. Appl. Physiol. *1*, 93–122.

Pinheiro, J., Bates, D., DebRoy, S., Sarkar, D., and R Core Team. (2019). nlme: Linear and Nonlinear Mixed Effects Models, R package version 3.1-141, https://CRAN.R-project.org/package=nlme.

Pinton, G., Aubry, J.-F., Bossy, E., Muller, M., Pernot, M., and Tanter, M. (2012). Attenuation, scattering, and absorption of ultrasound in the skull bone. Med. Phys. *39*, 299–307.

Pulkkinen, A., Huang, Y., Song, J., and Hynynen, K. (2011). Simulations and measurements of transcranial low-frequency ultrasound therapy: skull-base heating and effective area of treatment. Phys. Med. Biol. *56*, 4661–4683.

Raudenbush, S.W., and Bryk, A.S. (2001). Hierarchical Linear Models: Applications and Data Analysis Methods (SAGE Publications, Inc).

Sallet, J., Mars, R.B., Noonan, M.P., Neubert, F.-X., Jbabdi, S., O'Reilly, J.X., Filippini, N., Thomas, A.G., and Rushworth, M.F. (2013). The organization of dorsal frontal cortex in humans and macaques. J. Neurosci. *33*, 12255–12274.

San-Galli, A., Varazzani, C., Abitbol, R., Pessiglione, M., and Bouret, S. (2018). Primate Ventromedial Prefrontal Cortex Neurons Continuously Encode the Willingness to Engage in Reward-Directed Behavior. Cereb. Cortex *28*, 73–89.

Schemper, M., and Henderson, R. (2000). Predictive accuracy and explained variation in Cox regression. Biometrics *56*, 249–255.

Seo, H., and Lee, D. (2007). Temporal filtering of reward signals in the dorsal anterior cingulate cortex during a mixed-strategy game. J. Neurosci. *27*, 8366–8377.

Shadlen, M.N., and Kiani, R. (2013). Decision making as a window on cognition. Neuron *80*, 791–806.

Stoll, F.M., Wilson, C.R.E., Faraut, M.C.M., Vezoli, J., Knoblauch, K., and Procyk, E. (2016a). The Effects of Cognitive Control and Time on Frontal Beta Oscillations. Cereb. Cortex *26*, 1715–1732.

Stoll, F.M., Fontanier, V., and Procyk, E. (2016b). Specific frontal neural dynamics contribute to decisions to check. Nat. Commun. *7*, 11990.

Thaler, D., Chen, Y.C., Nixon, P.D., Stern, C.E., and Passingham, R.E. (1995). The functions of the medial premotor cortex. I. Simple learned movements. Exp. Brain Res. *102*, 445–460.

Turchi, J., Chang, C., Ye, F.Q., Russ, B.E., Yu, D.K., Cortes, C.R., Monosov, I.E., Duyn, J.H., and Leopold, D.A. (2018). The Basal Forebrain Regulates Global Resting-State fMRI Fluctuations. Neuron *97*, 940–952.e4.

Tustison, N.J., and Avants, B.B. (2013). Explicit B-spline regularization in diffeomorphic image registration. Front. Neuroinform. *7*, 39.

Verhagen, L., Gallea, C., Folloni, D., Constans, C., Jensen, D.E., Ahnine, H., Roumazeilles, L., Santin, M., Ahmed, B., Lehericy, S., et al. (2019). Offline impact of transcranial focused ultrasound on cortical activation in primates. eLife *8*, e40541.

Vincent, J.L., Patel, G.H., Fox, M.D., Snyder, A.Z., Baker, J.T., Van Essen, D.C., Zempel, J.M., Snyder, L.H., Corbetta, M., and Raichle, M.E. (2007). Intrinsic functional architecture in the anaesthetized monkey brain. Nature *447*, 83–86.

Wang, H., Kim, M., Normoyle, K.P., and Llano, D. (2016). Thermal Regulation of the Brain-An Anatomical and Physiological Review for Clinical Neuroscientists. Front. Neurosci. *9*, 528.

Wittmann, M.K., Kolling, N., Akaishi, R., Chau, B.K.H., Brown, J.W., Nelissen, N., and Rushworth, M.F.S. (2016). Predictive decision making driven by multiple time-linked reward representations in the anterior cingulate cortex. Nat. Commun. *7*, 12327.

Woolrich, M.W., Ripley, B.D., Brady, M., and Smith, S.M. (2001). Temporal autocorrelation in univariate linear modeling of FMRI data. Neuroimage *14*, 1370–1386.

Záborszky, L., Gombkoto, P., Varsanyi, P., Gielow, M.R., Poe, G., Role, L.W., Ananth, M., Rajebhosale, P., Talmage, D.A., Hasselmo, M.E., et al. (2018). Specific Basal Forebrain-Cortical Cholinergic Circuits Coordinate Cognitive Operations. J. Neurosci. *38*, 9446–9458.

Zhang, K., Chen, C.D., and Monosov, I.E. (2019). Novelty, Salience, and Surprise Timing Are Signaled by Neurons in the Basal Forebrain. Curr. Biol. *29*, 134–142.e3.

Zhao, X., Lynch, J.G., and Chen, Q. (2010). Reconsidering Baron and Kenny: Myths and Truths about Mediation Analysis. J. Consum. Res. *37*, 197–206.

STAR★METHODS

KEY RESOURCES TABLE

REAGENT or RESOURCE	SOURCE	IDENTIFIER
Chemicals, Peptides, and Recombinant Proteins		
Isoflurane – ISOFLO 250ml	Centaur	30135687
Ketamine – Narketan 10% 10ml INJ CD(SCH4)1 1-MCD	Centaur	03120257
Midazolam – Hypnoval amps 10mg/2ml	Centaur	23191407
Atropine – Atrocare INJ 25ml	Centaur	02500456
Meloxicam – Metacam INJ 10ml 5mg/ml DOGS/CATS	Centaur	02500456
Ranitidine 50mg/2ml x5 INJ	Centaur	30294115
Saline	DPAG, University of Oxford	N/A
Formalin	DPAG, University of Oxford	N/A
SignaGel Electrode Gel	Parker Laboratories	#15-25
Experimental Models: Organisms/Strains		
Macaca mulatta, 4 males, between 4-6 years old, between 11.6-14.2 kg, socially housed	MRC, Centre for Macaques	NCBITaxon:9544
Software and Algorithms		
MATLAB 2017a	Mathworks	N/A
Presentation	Neurobehavioral systems	N/A
FMRIB Software Library v5.0	FMRIB, WIN, Oxford, UK	N/A
Advanced Normalization Tools	Tustison and Avants, 2013	N/A
Connectome Workbench	The Human Connectome Project and Connectome Coordination Facility	N/A
Magnetic Resonance Comparative Anatomy Toolbox	Neuroecology Lab	https://github.com/neuroecology/MrCat
Offline_SENSE	Windmiller Kolster Scientific	N/A
R	The R Foundation	N/A
Mediation Toolbox	Cognitive Affective Neuroscience Laboratory	https://wagerlab.colorado.edu/tools
Other		
Transducer H-115MR 250kHz SN:018	Sonic Concepts	http://sonicconcepts.com
Transducer H-115MR 250kHz SN:017	Sonic Concepts	http://sonicconcepts.com
Amplifier Model 75A250A – 75Watts – 10khz 250MHz	Amplifier Research	https://www.arworld.us/
Tie Pie Handyscope HS5 SN: 32239	Tie Pie	https://www.tiepie.com/en
Brainsight frameless stereotaxic neuronavigation system	Rogue Research	N/A
MRI compatible frame	Crist Instruments	http://www.cristinstrument.com/products/stereotax/stereotax-primate
Four-channel phased-array coil	Windmiller Kolster Scientific	https://www.wkscientific.com/#mri-coils

LEAD CONTACT AND MATERIALS AVAILABILITY

Further information and requests for resources should be directed to and will be fulfilled by the Lead Contact, Nima Khalighinejad (nima.khalighinejad@psy.ox.ac.uk)

EXPERIMENTAL MODEL AND SUBJECT DETAILS

Four male rhesus monkeys (*Macaca mulatta*) were involved in the experiment. They weighed 11.6–14.2 kg and were 4-6 years of age. They were group housed and kept on a 12 hr light dark cycle, with access to water 12–16 hr on testing days and with free water access on non-testing days. All procedures were conducted under licenses from the United Kingdom (UK) Home Office in accordance with the UK The Animals (Scientific Procedures) Act 1986 and with the European Union guidelines (EU Directive 2010/63/EU).

METHOD DETAILS

Behavioral Training

Prior to the data acquisition, all animals were trained to work in an MRI compatible chair in a sphinx position that was placed inside a custom mock scanner simulating the MRI scanning environment. In the first stage of training, animals were trained to use custom-made infra-red touch sensors to respond to an image of a frame filled with dots that was presented either on the left or right side of the screen, with their left or right hand, respectively. They got a reward for touching the sensor on the side corresponding to the image within 4 s. The color of the image changed from trial-to-trial and animals learnt that different colors were associated with different levels of reward (drops of juice). In the second stage of training dots were presented gradually, one at a time. Animals learned to withhold their response for a few seconds before responding to increase their chance of getting a reward, given the probabilistic nature of the task. In the last stage of training animals learned to tolerate long action-outcome delays; given the hemodynamic lag in macaques (Chau et al., 2015) this delay allowed disambiguation of neural activity occurring at the time of decision making and at the time of decision outcome. The delay gradually increased from 0.5 s over several sessions to 4 s. The animals underwent aseptic surgery to implant an MRI compatible head post (Rogue Research, Mtl, CA). After a recovery period of at least 4 weeks, the animals were trained to perform the task inside the actual MRI scanner under head fixation. The imaging data acquisition started once they were receiving a reward on more than 50% of the trials (i.e., passing the midpoint of the probability distribution in more than half of the trials), for at least three consecutive sessions in the scanner.

Experimental task

At the beginning of each trial an empty frame (8 × 26 cm) appeared on the left or right side of the screen. The frame gradually filled with dots (round circles, r = 0.3 cm, max number of dots = 25) emerging from top to bottom (Figure 1B). Animals could terminate the trial, at a time of their own choice, by touching a custom-made infra-red touch sensor, on the side corresponding to the image. The trial continued if they touched the opposite side. The probability of getting reward increased as more dots appeared on the screen, following a sigmoid curve (Figure 1D). The probability distribution was drawn from a sigmoid function. The input to the function was a vector corresponding to the number of dots from 1 to 25. The midpoint of the curve was at dot #12 (50% chance of getting reward) with the steepness of 0.5. The probability distribution was constant across the trials and the sessions. The color of the frame and dots varied from trial to trial but remained constant within a trial. The color indicated potential reward magnitude and could be red, green or blue, indicating one, two or three drops of juice, respectively. In addition to the color, the speed of the dots appearance also varied from trial to trial. A new dot appeared every 100, 200 or 300 ms. Animals had the option to respond, any time from the beginning of the trial (appearance of the empty frame) to 300 ms after the frame was filled (appearance of the last dot). If they responded, they were offered drops of juice or no juice, based on the probability distribution at the time of response. There was a delay of 4 s between response and outcome (action-outcome delay). Successful and unsuccessful outcomes were indicated by an upward and downward pointing triangle, respectively. The triangle remained on the screen for 2 s. If rewarded, drops of blackcurrant juice were delivered by a spout placed near the animal's mouth during scanning. Each drop was composed of 1 mL blackcurrant juice. No juice was delivered when the trial was not rewarded. After the outcome phase, they proceeded to the next trial after a 3, 5 or 7 s inter-trial interval (ITI). ITI varied in blocks of 30 trials in a pseudo-randomized order. Specific patterns on the left and right side of the screen indicated the ITI block (Figure 1C). If animals did not respond by 300ms after the emergence of the last dot, the frame disappeared, and they had to wait for 4 s (equivalent to action-outcome delay) + 3, 5 or 7 s (ITI) for the next trial to start. Animals were given 40min to perform the task at each session. The task finished after 40 min, regardless of the number of trials done. Each animal performed ten to twelve sessions in the MRI scanner. The experiment was controlled by Presentation software (Neurobehavioral Systems Inc., Albany, CA).

Cox regression model

To estimate the deterministic component of actTime we used a specific class of survival models called the Cox proportional hazard model (Murakami et al., 2017). The model predicts time-to-event (*actTime*) on the current trial from present and past contextual factors. Specifically, the predictors (covariates) included reward magnitude, dot speed and ITI of the current trial, and the actual reward and *actTime* on the past 10 trials. Importantly, actTime in trials that monkeys decided not to respond are labeled as 'censored' data because in those trials monkeys might have responded if the trials were to continue for longer. The model is described as:

$$\lambda(t) = \lambda_0(t).\exp(\beta\mathbf{x}),$$

where $\lambda(t)$represents a hazard function (hazard rate of responding), $\lambda_0(t)$ represents a baseline hazard function, that is a hazard function when all the covariates are 0, β is a row vector with 23 elements (3 present contextual factors + 10 past rewards + 10 past actTimes) representing Cox coefficients for each covariate and **x** is a 23 element column vector representing covariates, present contextual factors and contextual factors of the past 10 trials. The coefficients were estimated for each testing session by using the 'coxphfit' function in MATLAB. We also investigated an alternative model where we introduced 'trial number' as a covariate to account for a potential effect of satiety on actTime; however, the Cox coefficient for trial number was not significantly different from zero. Moreover, its inclusion had a negligible impact on the proportion of variance in *actTime* explained by the other factors. This may partly reflect the relatively short duration of each testing session.

A detailed method for obtaining Cox coefficients has been previously described (Murakami et al., 2017). The estimated Cox coefficients $(\widehat{\beta})$ from present and past contextual factors (only coefficients from the immediately preceding trial were used, as only these had been significant for both past reward outcome and past *actTime*) were used to obtain the expected *actTime* from the given predictors by the following method:

First, the cumulative hazard function, $\widehat{\Lambda}_x(t)$, of each trial was estimated given the baseline cumulative hazard function, $\widehat{\Lambda}_0(t)$, and the covariates:

$$\widehat{\Lambda}_x(t) = \widehat{\Lambda}_0(t).\exp\left(\widehat{\beta}\mathbf{x}\right),$$

The cumulative hazard function of each trial was then used to estimate the survival function of each trial, *S(t)*:

$$\widehat{S}_x(t) = \exp\left(-\widehat{\Lambda}_x(t)\right),$$

The deterministic *actTime* is estimated by:

$$[actTime] = \int_0^\infty \widehat{S}_x(t),$$

We also separately assessed the contribution of past and present context to deterministic *actTime*. The original model was split into two, estimating the Cox regression coefficients separately from its present and past components. We then used the Cox coefficients relating to either the present or the immediately preceding trial (similar to the original model) to derive two separate *actTime* estimates. These new estimates were termed *deterministic* $actTime_{present\ context}$ and *deterministic* $actTime_{past\ context}$.

Finally, to measure the proportion of variance explained by the Cox regression model, we used Schemper's *V* (Schemper and Henderson, 2000), which is defined as:

$$V = \frac{\left(\widehat{D} - \widehat{D}_x\right)}{\widehat{D}},$$

Where $\widehat{D}$ is the distance between survival functions of individual trials $S_i(t)$ and a survival function estimated from all the trials without taking into account covariates $\widehat{S}(t)$, by using Kaplan–Meier estimator.$\widehat{D}_x$ is calculated in the same way as $\widehat{D}$, but is the distance between survival functions of individual trials $S_i(t)$, and an estimated conditional survival function given covariates $\mathbf{x}$, $\widehat{S}_x(t)$. The equations to calculate $\widehat{D}$ are previously described in detail (Murakami et al., 2017).

Imaging data acquisition

Awake-animals were head-fixed in a sphinx position in an MRI-compatible chair (Rogue Research, MTL, CA). MRI was collected using a 3T horizontal bore MRI clinical scanner and a four-channel phased array receive coil in conjunction with a radial transmission coil (Windmiller Kolster Scientific Fresno, CA). Each loop of the coil had an 8cm diameter which ensures a good coverage of the animal's head. Similar coils have been previously used for awake fMRI studies in primates (Chau et al., 2015; Kolster et al., 2014; Papageorgiou et al., 2017). The chair was positioned on the sliding bed of the scanner. The receiver coils were placed on the side of the animal's head with the transmitter placed on top. The touch sensors and the juice delivery system were the same as the one used in the mock scanner. An MRI-compatible screen (MRC, Cambridge) was placed 30cm in front of the animal and the image was projected on the screen by a LX400 projector (Christie Digital Systems). Functional data were acquired using a gradient-echo T2* echo planar imaging (EPI) sequence with a 1.5 × 1.5 × 1.5 mm resolution, repetition time (TR) 2.28 s, echo time (TE) 30 ms and flip angle 90°. At the end of each session, proton-density-weighted images were acquired using a gradient-refocused echo (GRE) sequence with a 1.5 × 1.5 × 1.5 mm resolution, TR 10 ms, TE 2.52 ms, and flip angle 25°. These images were later used for offline MRI reconstruction. T1-weighted MP-RAGE images with a resolution of 0.5 × 0.5 × 0.5 mm, TR 2.5 s, TE 4.04 ms, inversion pulse time (TI) 1.1 s, and flip angle 8°, were acquired in separate sessions under general anesthesia. Anaesthesia was induced by intramuscular injection of 10 mg/kg ketamine, 0.125-0.25 mg/kg xylazine, and 0.1 mg/kg midazolam and maintained with isoflurane (for details see Sallet et al., 2013). Anaesthesia was only used for collecting T1-weighted structural images.

fMRI data preprocessing

Preprocessing was performed using tools from FMRIB Software Library (FSL) (Jenkinson et al., 2012), Advanced Normalization Tools (ANTs; http://stnava.github.io/ANTs) (Tustison and Avants, 2013), Human Connectome Project Workbench (Glasser et al., 2013) (https://www.humanconnectome.org/software/connectome-workbench), and the Magnetic Resonance Comparative Anatomy Toolbox (MrCat; https://github.com/neuroecology/MrCat). First, T2* EPI images acquired during task performance were reconstructed by an offline-SENSE method that achieved higher signal-to-noise and lower ghost levels than conventional online reconstruction (Kolster et al., 2009) (Offline_SENSE GUI, Windmiller Kolster Scientific, Fresno, CA). A low-noise EPI reference image was created for each session, to which all volumes were non-linearly registered on a slice-by-slice basis along the phase-encoding

direction to correct for time-varying distortions in the main magnetic field due to body and limb motion. The aligned and distortion-corrected functional images were then non-linearly registered to each animal's high-resolution structural images. A group specific template was constructed by registering each animal's structural image to the CARET macaque F99 space (Kolster et al., 2009). Finally, the functional images were temporally filtered (high-pass temporal filtering, 3-dB cutoff of 100 s) and spatially smoothed (Gaussian spatial smoothing, full-width half maximum of 3mm).

Transcranial Ultrasound Stimulation (TUS)

Ultrasound stimulation was performed using a single element ultrasound transducer with 63.2 mm radius of curvature (H115-MR, diameter 64 mm, Sonic Concept, Bothell, WA, USA). The transducer was coupled with a coupling cone and was filled with degassed water and sealed with a latex membrane (Durex). The ultrasound wave frequency was set to 250 kHz resonance frequency. 30 ms bursts of ultrasound were generated every 100 ms with a digital function generator (Handyscope HS5, TiePie engineering, Sneek, the Netherlands) for a total duration of 40 s. A 75-Watt amplifier (75A250A, Amplifier Research, Souderton, PA) was used to deliver the required power to the transducer. A TiePie probe connected to an oscilloscope was used to monitor the output voltage. The recorded peak-to-peak voltage was constant throughout the stimulation session and ranged from 128 to 136 V. This corresponds to a peak negative pressure of 1.152 to 1.292 MP, respectively, measured in water with an in-house heterodyne interferometer (see reference Constans et al., 2017 for more details about the calibration protocol).

At the beginning of each stimulation session the animal's skull was shaved and a conductive gel (SignaGel Electrode; Parker Laboratories Inc.) was applied to the skin. The water-filled coupling cone and the gel was used to ensure ultrasonic coupling between the transducer and the animal's head. Next, the ultrasound transducer / coupling cone montage was placed on the skull and a Brainsight Neuronavigation System (Rogue Research, Montreal, CA) was used to position the montage so that the focal spot would be centered on the targeted brain region. Ad hoc coupling cone were used for each target of interest. All targets were sonicated bilaterally for 80 s in total, with 40 s of stimulation applied to a target in each hemisphere. Sonication of the target in one hemisphere was immediately followed by sonication of a homologous target in the contralateral hemisphere. Hemispheres were sonicated in a pseudo-random order. After stimulation, monkeys were immediately moved to a testing room for behavioral data collection. There were four stimulation conditions (Figure 7C): ACC, BF, parietal cortex and sham. Left and right ACC and BF targets were defined based on the whole-brain peak activity for *actTime* contrast, projected on each individual monkey's structural image. We targeted amBF where we had strong bilateral activity; however, as explained in the Results, the stimulation was also associated with activity change in adjacent plBF. Left and right posterior parietal operculum (POp) targets were used as active control stimulation sites. A sham condition was also implemented as a non-stimulation passive control. The sham condition completely matched a typical stimulation session (setting, stimulation procedure, neuro-navigation, targeting, transducer preparation and timing of its bilateral application to the shaved skin on the head of the animal) except that sonication was not triggered. During the sham session the montage was pseudo-randomly positioned to target ACC, BF or POp. Each stimulation condition was repeated five times, on separate days, and the order of the stimulation sessions was pseudo-randomized for each animal. For example, the stimulation schedule for monkey W was POp TUS – sham TUS – ACC TUS – BF TUS, repeated five times, over 40 days. The stimulation was always performed at the same time of the day and there was always a 48 hours gap between each session, regardless of it being a real or sham stimulation session.

The TUS procedure used here, in which a short train of TUS was delivered, has a short-term impact on neural activity and behavior that lasts many minutes to a few hours (Folloni et al., 2019; Fouragnan et al., 2019; Verhagen et al., 2019). This ensures that neither the neural nor the behavioral effects of TUS found here can be attributed to the auditory stimulation that accompanies TUS delivery (Airan and Butts Pauly, 2018; Mohammadjavadi et al., 2019).

Acoustic and thermal modeling

The acoustic wave propagation of our focused ultrasound protocol was simulated using a k-space pseudospectral method-based solver, k-Wave (Cox et al., 2007) to obtain estimates for the pressure amplitude, peak intensity, spatial distribution, and thermal impact at steady state. 3D maps of the skull were extracted from a monkey CT scan (monkey L (Constans et al., 2017), 0.14 mm slice resolution, 0.33 mm slice distance). Soft tissues were assumed to be homogeneous, with acoustic values of water (ρ_{tissue} = 1000 kg.m^{-3} and c_{tissue} = 1500 m.s^{-1}). In the bone, a linear relationship between the Hounsfield Units (HU) from the CT scan and the sound speed, as well as the density, was used. The power law model for attenuation is $\alpha_{att} = \alpha_{min} + \alpha_{max} * \Phi^{\beta}$ where the porosity Φ is defined by $\Phi = (\rho_{max} - \rho / \rho_{max} - \rho_{tissue})$in the skull (Aubry et al., 2003). The attenuation coefficients for the acoustic propagation α_{min} and α_{max}depend on the frequency: $\alpha_{min} = \alpha_{min0} f^{b}$with $\alpha_{min0} = 0.2$ dB.cm^{-1}.MHz^{-b} and $\alpha_{max} = \alpha_{max0} f^{b}$with $\alpha_{max0} = 8$ dB.cm^{-1}.MHz^{-b} (Aubry et al., 2003). We set the parameters to ρ_{max} = 2200 kg.m^{-3}, c_{max} = 3100 m.s^{-1}(Constans et al., 2017), $\beta = 0.5$ (Aubry et al., 2003), b = 1.1 (Constans et al., 2018). The attenuation coefficient in bone accounts for both absorption and scattering (Pinton et al., 2012).

The propagation simulation was performed at 250 kHz with a 150 µs-long pulse signal (enough to reach a steady state). The transducer was modeled as a spherical section (63.2 mm radius of curvature and 64 mm active diameter). The simulated pulses were spatially apodized (r = 0.35) on the spherical section. Ultrasound propagates first through water before entering the skull cavity

with the geometrical focal point located below the surface, inside the brain. Simulations were performed in free water, and the maximum amplitude obtained was used to rescale the results in skull. The thermal modeling is based on the bio-heat equation (Pennes, 1948):

$$\rho C \frac{\partial T}{\partial t} = \kappa \nabla^2 T + q + w\rho_b C_b (T - T_a)$$

where T, ρ, C, κ and q are the temperature, density, specific heat, thermal conductivity and rate of heat production respectively. Heat production is defined as $q = \alpha_{abs}(P^2 / 2\rho C)$, α_{abs} being the absorption coefficient and P the peak negative pressure. κ is set to 0.528 $W.m^{-1}.K^{-1}$ in soft tissue and 0.4 $W.m^{-1}.K^{-1}$ in the skull; C is set to 3600 $J.kg^{-1}.K^{-1}$ in soft tissue and 1300 $J.kg^{-1}.K^{-1}$ in the skull (Duck, 2012). In the tissue, the absorption coefficient was set to $\alpha_{abs\ tissue} = 0.21$ $dB.MHz^{-b}$ (Goss et al., 1979). In the skull the longitudinal absorption coefficient is proportional to the density with $\alpha_{abs\ max} = (\alpha_0 / 3) = 2.7$ $dB.cm.MHz^{-b}$ (Pinton et al., 2012). The last term corresponds to the perfusion process: $w, \rho_{b,} C_b$ and T_a correspond to the blood perfusion rate, blood density, blood specific heat and blood ambient temperature respectively. These parameters are assumed homogeneous over the brain, although a more detailed description of the brain cooling processes can be found in the literature (Wang et al., 2016). The perfusion parameters are based on previous reports (Pulkkinen et al., 2011): w = 0.008 s^{-1}; ρ_b = 1030 $kg.m^{-3}$; C_b = 3620 $J.kg^{-1}.K^{-1}$ and T_a = 37°C.

The bioheat equation is solved by using a 3D finite-difference scheme in MATLAB (Mathworks, Natick, USA) with Dirichlet boundary conditions. Initial temperature conditions were 37°C in the brain, skull and tissue, and 24°C in the water coupling cone. Simulations were run over 1 minute pre-sonication, followed by 40 s of sonication, and 5 minutes post-sonication, closely following the experimental procedure.

Resting-state imaging data acquisition, pre-processing, and analysis

The detailed procedure for rs-fMRI acquisition, pre-processing, and analysis has been reported elsewhere (Folloni et al., 2019; Verhagen et al., 2019). In summary, rs-fMRI was collected under inhalational isoflurane gas anesthesia for three monkeys after BF TUS, SMA TUS (Verhagen et al., 2019), and a control state (Verhagen et al., 2019). During the acquisition of rs-fMRI data the mean expired isoflurane concentration was around 1%. Isoflurane was chosen to maintain anesthesia as it has been previously demonstrated to preserve rs-fMRI networks (Mars et al., 2013; Neubert et al., 2015; Sallet et al., 2013; Vincent et al., 2007). Moreover, the impact of the transcranial ultrasound stimulation (TUS) was established by comparing rs-fMRI sessions collected after TUS with sessions without prior TUS but in both cases data were collected under identical conditions of isoflurane anesthesia. Because animals were anaesthetised in both cases it is therefore possible to compare the impact of TUS stimulation on functional connectivity of the stimulated areas. After pre-processing, we used a seed-based correlation analysis approach (Neubert et al., 2015; Sallet et al., 2013) to report the whole-brain functional connectivity of the stimulation site (BF) with and without TUS. Additionally, we reported whole-brain functional connectivity for SMA and POp with BF TUS, SMA TUS and no stimulation (Figure S6). The impact of BF TUS was quantified by regressing, for each point in the brain, the seed-based correlation map observed after BF TUS against the seed-based correlation map observed in the control state (Figure 7B).

QUANTIFICATION AND STATISTICAL ANALYSIS

Behavioral analysis

Time to act (*actTime*) was defined as the number of dots on the screen at the time of response. We used a multilevel generalized linear model (GLM) to predict *actTime* from present and past contextual factors, with ‘trial data’ as level one variable, ‘testing session’ as level two and ‘animal’ as level three. The variables ‘testing session’ and ‘animal’ were assigned as the random part of the model, with the variable ‘testing session’ nested within the variable ‘animal’. Note that by-subject random slopes were not included in the multilevel models, because it is suggested that a random variable should have at least 10 levels before one can include random effects for it (sample size in this study was four) (Raudenbush and Bryk, 2001). Maximum likelihood method was used for model estimation and pairwise t test and Tukey’s HSD for post hoc comparisons. We examined the impact of both present and past contextual factors on *actTime*. Present contextual factors consisted of potential reward magnitude, speed of dots and ITI on the current trial. Past contextual factors consisted of actual reward outcome and *actTime* on the past trial.

$$actTime_t = \beta_0 + \beta_1 reward_t + \beta_2 dotSpeed_t + \beta_3 ITI_t + \beta_4 (reward_t * dotSpeed_t) + \beta_5 (reward_t * ITI_t) + \beta_6 (dotSpeed_t * ITI_t) + \beta_7 (reward_t * dotSpeed_t * ITI_t) + \beta_8 rewardOutcome_{t-1} + \beta_9 actTime_{t-1}$$

Likewise, we used a hierarchical logistic regression to predict the odds of responding at a given trial from contextual factors.

$$logit\ (response_t) = \beta_0 + \beta_1 reward_t + \beta_2 dotSpeed_t + \beta_3 ITI_t + \beta_4 rewardOutcome_{t-1} + \beta_5 actTime_{t-1}$$

The modeling was performed by ‘nlme’ and ‘lme4’ packages in R (Bates et al., 2018; Pinheiro et al., 2018).

fMRI data analysis

To perform whole-brain statistical analyses we used a univariate generalized linear model (GLM) framework as implemented in FSL FEAT (Woolrich et al., 2001). At the first level, we constructed a GLM to compute the parameter estimates (PEs) for each regressor. The GLM was constructed based on the linear model we previously used for behavioral analyses:

$$GLM1: BOLD = \beta_0 + \beta_1\ resp + \beta_2\ reward + \beta_3\ dotSpeed + \beta_4\ ITI + \beta_5\ pastRew + \beta_6\ pastactTime + \beta_7\ actTime + \beta_8\ time + \beta_9\ rightconv + \beta_{10}\ leftconv + \beta_{11}\ mainOut + \beta_{12}\ levelOut + \beta_{13}\ rightunconv + \beta_{14} leftunconv + \beta_{15}\ mouth,$$

where *BOLD* is a t x 1 (t time samples) column vector containing the times series data for a given voxel. Trials where animals made no response were left out of the analysis (16.38% ± 11.42% of the trials, across all sessions). Regressors 1 to 7 are task-related regressors of interest: *resp* is an unmodulated regressor representing the main effect of stimulus presentation in responded trials (all event amplitudes set to one). *reward, dotSpeed* and *ITI* are parametric regressors with three levels, which represent reward magnitude, speed of dots, and inter-trial-interval on the current trial, respectively. *pastRew* is a parametric regressor with four levels representing the reward outcome on the past trial. *pastactTime* is also parametric and represents *actTime* on the past trial. *pastRew* and *pastactTime* were both weighted by their influence on *actTime* on the current trial (multiplied by their coefficients from behavioral GLM). *actTime* represents time-to-act (number of dots at response) on the current trial. Regressors 1 to 7 were all boxcar regressors with a duration of 500 ms that were convolved with a hemodynamic response function (HRF) specific for monkey brains (Kagan et al., 2010; Nakahara et al., 2002). Regressors 1-6 were all time-locked to the onset of the trial. Regressor 7 started 500 ms before animals made a response by cutting the infra-red touch sensor and continued for 500 ms.

Regressors 8-15 were task-related confound regressors. *time* is a parametric regressor representing the time passed since the beginning of the scanning session and is locked to the trial onset. *leftconv* and *rightconv* are unmodulated regressors (all event amplitudes set to one), locked to 500 ms prior to response, representing the response with the left and right hand, respectively. *mainOut* is an unmodulated regressor representing the main effect of outcome (all event amplitudes set to one). *levelOut* is a parametric regressor with four levels representing the reward outcome on the current trial. Regressors 11-12 were locked to the onset of outcome (juice) delivery. Regressors 13-15 modeled instant signal distortions due to changes in the magnetic field caused by movement. These regressors were therefore not convolved with HRF. *rightunconv* and *leftunconv* represented distortion due to right and left-hand responses. They started at the beginning of the TR when the response was recorded and had a duration of one TR (2.28 s). *mouth* represented distortion due to mouth movements. It started at the beginning of the TR when the juice delivery started and terminated at the end of the TR when the juice delivery ended. To further reduce variance and noise in the BOLD signal, we also added task-unrelated confounds which included 13 parametric PCA components that describe, for each volume, the warping from that volume to the mean reference image when correcting motion artifacts.

First level analysis was performed on each scanning session (45 sessions in total). The contrast of parameter estimates (COPEs) and variance estimates (VARCOPEs) from each scanning session were then combined in a second-level mixed-effects analysis (FLAME 1+2) treating sessions as random effects. Time series statistical analysis was carried out using FMRIB's improved linear model with local autocorrelation correction.

ROI analysis

Regions of interest (ROI) were defined as spheres of 3 mm radius, centered at the peak of the activation of a contrast. For time-series analyses, the filtered time-series of each voxel within each ROI was averaged, normalized and up-sampled. The up-sampled data was then epoched in 8 s windows, starting from 2 s before to 6 s after the response time. Time-series GLMs were then fit at each time step of the epoched data, in responded trials, using ordinary least-squares (OLS). We ran the following GLMs:

GLM2.1

$$BOLD = \beta_0 + \beta_1\ actTime_{numDot} + \beta_2\ time + \beta_3\ nontask,$$

where *BOLD* is a *i x t* (*i* trial, *t* time samples) matrix containing the times series data for a given ROI. $actTime_{numDot}$ is the number of dots at response. *time* is the time passed since the beginning of the testing session. *nontask* is a task-unrelated unmodulated constant regressor.

GLM2.2

$$BOLD = \beta_0 + \beta_1\ actTime_{numDot} + \beta_2\ actTime_{second} + \beta_3\ time + \beta_4\ nontask,$$

where $actTime_{seconds}$ is the log time-to-act in seconds.

GLM2.3

$$BOLD = \beta_0 + \beta_1\ deterministic\_actTime_{present+past} + \beta_2\ actTime_{numDot} + \beta_3\ actTime_{second} + \beta_4\ time + \beta_5\ nontask,$$

where $deterministic\_actTime_{present+past}$ is the number of dots at response as predicted by the Cox regression model from present and past contextual factors.

GLM2.4

$$BOLD = \beta_0 + \beta_1 deterministic\_actTime_{present} + \beta_2\ deterministic\_actTime_{past} + \beta_3\ actTime_{numDot} + \beta_4\ actTime_{second} + \beta_5\ time + \beta_6\ nontask,$$

where $deterministic\_actTime_{present}$ is the number of dots at response as predicted by the Cox regression model from present contextual factors and $deterministic\_actTime_{past}$ is the number of dots at response as predicted by the Cox regression model from past contextual factors.

GLM2.5

$$BOLD_{ROI} = \beta_0 + \beta_1\ BOLD_{seed} + \beta_2\ deterministic\_actTime_{present+past} + \beta_3\ PPI + \beta_4\ actTime_{numDot} + \beta_5\ actTime_{second} + \beta_6\ time + \beta_7\ nontask,$$

where $BOLD_{ROI}$ is BOLD activity at amBF, $BOLD_{seed}$ is BOLD activity at plBF, and PPI is the interaction between $BOLD_{seed}$ and $deterministic\_actTime_{present+past}$.

GLM2.6

$$BOLD_{ROI} = \beta_0 + \beta_1\ BOLD_{seed} + \beta_2\ quadReward + \beta_3\ PPI + \beta_4\ time + \beta_5\ nontask,$$

where $BOLD_{ROI}$ is BOLD activity at ACC, $BOLD_{seed}$ is BOLD activity at plBF, $quadReward$ is the quadratic reward magnitude, and PPI is the interaction between $BOLD_{seed}$ and $quadReward$.

Leave one out on time-series group peak signal

Significance testing on time-course data was performed by using a leave-one-out procedure on the group peak signal to avoid potential temporal selection biases. For every session, we calculated the time course of the group mean beta weights of the relevant regressor based on the remaining 44 sessions. We then identified the (positive or negative) group peak of the regressor of interest within the full width of the epoched time course: from 2 s before to 6 s after the response. Next, we took the beta weight of the remaining session at the time of the group peak. We repeated this for all sessions. Therefore, the resulting 45 peak beta weights were selected independently from the time course of each single session. We assessed significance using multilevel ANOVA and t tests on the resulting beta weights. The variables 'testing session' and 'animal' were assigned as the random part of the model, with the variable 'testing session' nested within the variable 'animal'. Note that to avoid any circularity in analyses, the regressors that first used to identify the locations of ROIs were always used as covariates in subsequent analyses of other signals extracted from those ROIs.

Mediation analysis

To investigate the mechanisms that underlie the relationship between contextual factors and *actTime* we performed multi-level mediation analysis using the Mediation Toolbox (https://wagerlab.colorado.edu/tools). We asked whether covariance between an initial variable (contextual factors) and an outcome variable (*actTime* as measured by number of dots) can be explained by a mediator variable (BOLD activity from ROI). Mediation analysis extends the previous univariate model and would allow us to test four effects (Figure 6A): the direct effect of initial variable (e.g., reward) on outcome variable (*actTime*) (path *c*); impact of initial variable on brain activity (path *a*); the impact of brain activity on outcome variable, controlling for initial variable (path *b*); and mediation of initial variable on outcome variable by a potential mediator (BOLD signal) (path *a x b*). The mediator variable was defined as the positive peak of the BOLD signal within the full width of the epoched time course for each ROI.

Significance estimates for paths *a*, *b, c,* and the mediation path were computed by bootstrapping using 10000 samples with replacement. The only requirement to demonstrate mediation, when using bootstrapping, is a significant indirect effect (path *a x b*) (Hayes, 2018; Zhao et al., 2010). A significant mediation suggests that including the brain activity in the path between contextual factors and *actTime*, significantly alters the slope of their relationship. P values were calculated for each ROI from the bootstrap confidence intervals. As in previous analyses, the log time-to-act in seconds and the time passed since the beginning of the session were added to the model as covariates of no interest.

TUS behavioral data analysis

Based on our previous findings, we designed two hypothesis-driven multilevel GLMs to investigate the effect of ACC TUS on the relationship between reward magnitude and observed *actTime* (GLM3.1), and the effect of BF TUS on the relationship between deterministic and observed *actTime* (GLM3.2). In both models, the variables 'stimulation session' and 'animal' were assigned as the random part of the model, with the variable 'stimulation session' nested within the variable 'animal'.

GLM3.1

$$actTime_t = \beta_0 + \beta_1 ROI\ TUS_t + \beta_2 reward_t + \beta_3 (reward_t * ROI\ TUS_t),$$

Where *ROI TUS* is the stimulation target and *reward* is the reward magnitude at trial *t*. To find out how stimulation targets might differ from each other without inflating type I error rate, ANOVA was followed by planned contrasts where the effect of ACC TUS on the relationship between reward magnitude and observed *actTime* was compared with the effects of BF, control POp and sham TUS.

GLM3.2

$$actTime\ bias_t = \beta_0 + \beta_1 ROI\ TUS_t,$$

Where *ROI TUS* is the stimulation target and $actTime\ bias_t$ is the absolute difference between observed and deterministic *actTime* at trial *t:*

$$|observed\ actTime_t - deterministic\ actTime_t|.$$

To estimate deterministic *actTime*, we first asked whether present and past contextual factors significantly contribute to the model. The Cox regression coefficients were significant for present (reward magnitude, dot speed, and ITI) and past (reward outcomes and past *actTimes*) contextual factors. However, compared to the first Cox model used to analyze the fMRI data, coefficients from the past trials were significant as far as 8 (past reward outcomes) and 9 (past *actTime*) trials back. Therefore, to increase the estimation accuracy, we used the Cox regression coefficients from all past 10 trials to estimate deterministic *actTime* from present and past contextual factors. ANOVA was followed by planned contrasts where the effect of BF TUS on *actTime bias* was compared with the effects of ACC, control POp and sham TUS.

DATA AND CODE AVAILABILITY

The data that support the findings of this study and the code to generate the results and the figures are available from the Lead Contact upon request.

Neuron, Volume *105*

Supplemental Information

A Basal Forebrain-Cingulate Circuit

in Macaques Decides It Is Time to Act

Nima Khalighinejad, Alessandro Bongioanni, Lennart Verhagen, Davide Folloni, David Attali, Jean-Francois Aubry, Jerome Sallet, and Matthew F.S. Rushworth

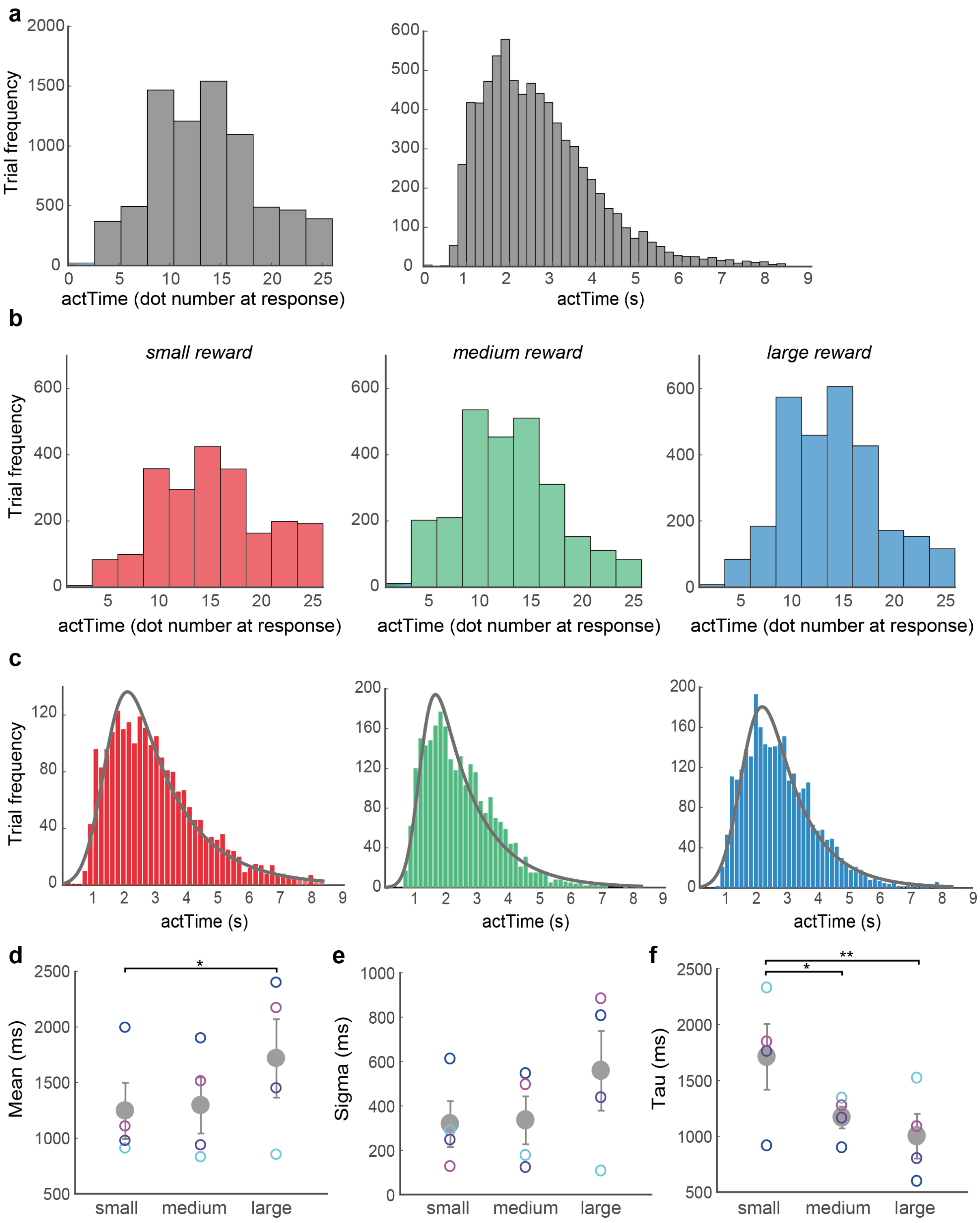
a
Trial frequency
actTime (dot number at response)
actTime (s)
b
small reward
medium reward
large reward
Trial frequency
actTime (dot number at response)
c
Trial frequency
actTime (s)
d
Mean (ms)
e
Sigma (ms)
f
Tau (ms)
small
medium
large

Figure S1. Distribution of *actTime*. Related to Figure 2. (a) Distribution of *actTime* across all trials. The histogram shows a wide range of *actTime* in dot number (left panel) and seconds (right panel); while on some trials animals were impulsive and responded after emergence of the first 2-3 dots, on many trials they waited till the very last dot before responding. (b) To get a better understanding of the U-shaped relationship between reward magnitude and *actTime* we looked at the distribution of *actTime* for different levels of reward. The histograms suggest that despite the quadratic effect of reward magnitude on *actTime*, the distribution of *actTime* has a distinct pattern in small and large reward trials: *actTimes* are distributed more uniformly in the former compared to the latter. (c) To capture the extended tail of the time-to-act distribution we fitted an exponentially modified Gaussian distribution on each animal's time-to-act in milliseconds, separately for small, medium and large reward magnitude trials. The ex-Gaussian model is derived via convolution of an exponential distribution and a normal distribution and captures response time distributions well (Lewandowsky and Farrell, 2011). It assumes that response time can be divided into two independent components: 1) the time taken to make a decision, assumed to be exponentially distributed (Andrews and Heathcote, 2001; Balota et al., 2008), and 2) processes supplementary to the decision assumed to be Gaussian (Luce, 1991). (d-f) The model has three free parameters: mean and standard deviation (sigma) of the Gaussian distribution and tau, the rate of drop-off of the exponential distribution. We found a significant main effect of reward magnitude on the mean ($X^2(2)=7.51$, $P=0.023$) (d), but not on the standard deviations of the Gaussian components ($X^2(2)=3.68$, $P=0.16$) (e). However, tau was significantly higher in small reward compared to large reward magnitude trials (main effect, $X^2(2)=12.48$, $P=0.002$; small vs. large, $\beta=711\pm156$, $t(6)=4.55$, $P=0.004$; small vs. medium, $\beta=543\pm156$, $t(6)=3.47$, $P=0.013$) (f). This suggests animals made more deliberate decisions when offered large compared to small rewards; decisions about when to act were relatively tightly clustered around long *actTimes*. However, when confronted with small rewards, animals appeared relatively indifferent and there was considerable variation in *actTime*, sometimes waiting till the very last moment and responding just before the trial

ended. Error bars represent standard error of the mean, and the grey circles illustrate the group mean. The model was separately fitted on pooled data (time-to-act in milliseconds) from each animal. Fitting was performed in MATLAB (MathWorks, MA, USA) by using maximum likelihood estimation and a bounded Simplex algorithm. Each colour ring represents the best fit for each animal. Multilevel ANOVA followed by pairwise t-test. * $P<0.05$, ** $P<0.01$, *** $P<0.001$.

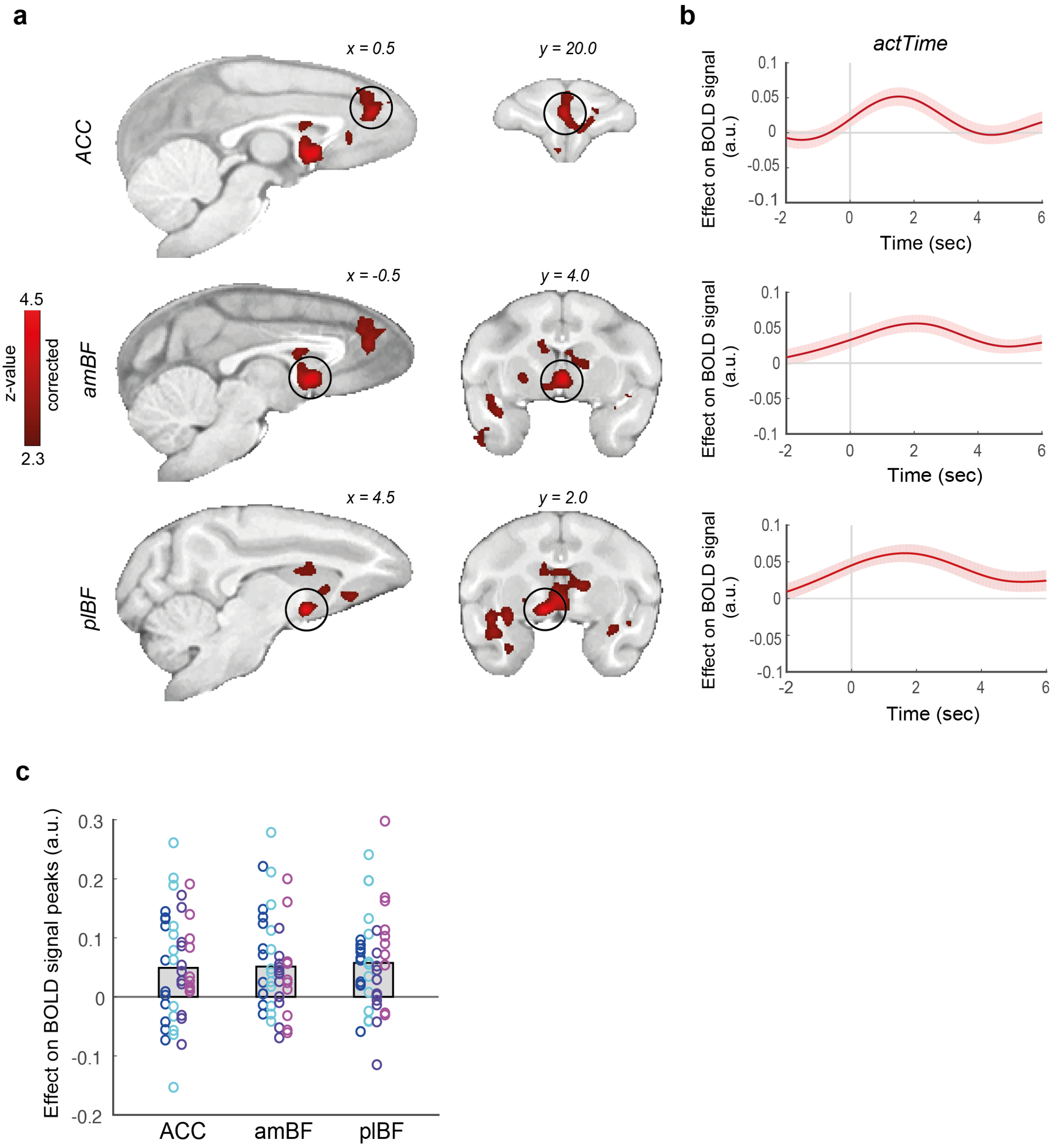

a
b
c
x = 0.5
y = 20.0
ACC
x = -0.5
y = 4.0
amBF
x = 4.5
y = 2.0
plBF
4.5
z-value
corrected
2.3
actTime
Effect on BOLD signal (a.u.)
Time (sec)
Effect on BOLD signal peaks (a.u.)
ACC
amBF
plBF

Figure S2. ACC and BF encode time to act. Related to Figure 4. (a) Whole-brain analysis showing voxels where activity reflected parametric variation in the empirically observed *actTime*, as indexed by dot number at the time of response. The analysis was performed in the same manner as in Fig.4a. but here the GLM only included *actTime* and not all the present and past contextual factors that influenced *actTime*. Top panel; ACC. Middle panel; amBF. Bottom panel; plBF. Whole-brain cluster-based correction, Z>2.3. (b) ROI time-course analysis of the ACC (top panel), amBF (middle panel), and plBF (bottom panel), showing the relationship between BOLD activity and parametric variation in *actTime*, after adding time-to-act in seconds to GLM2.1 (Methods) as a covariate to control for passage of time (Methods, GLM2.2). The lines and shadings show the mean and standard error of the β weights across the sessions, respectively. Time zero is the response time. Note that due to delay in the BOLD hemodynamic response function the BOLD signal time-course peaks 3s after neural activity. Once the delay in BOLD response is taken into account it is clear that BOLD activity reflects neural events occurring before the response onset. (c) No significant difference in *actTime* encoding was observed between ACC, amBF and plBF after adding time-to-act in seconds to the GLM as a covariate to control for passage of time. Each colour represents one animal, and each ring is the peak beta-weight of one testing session. The grey columns illustrate the group mean.

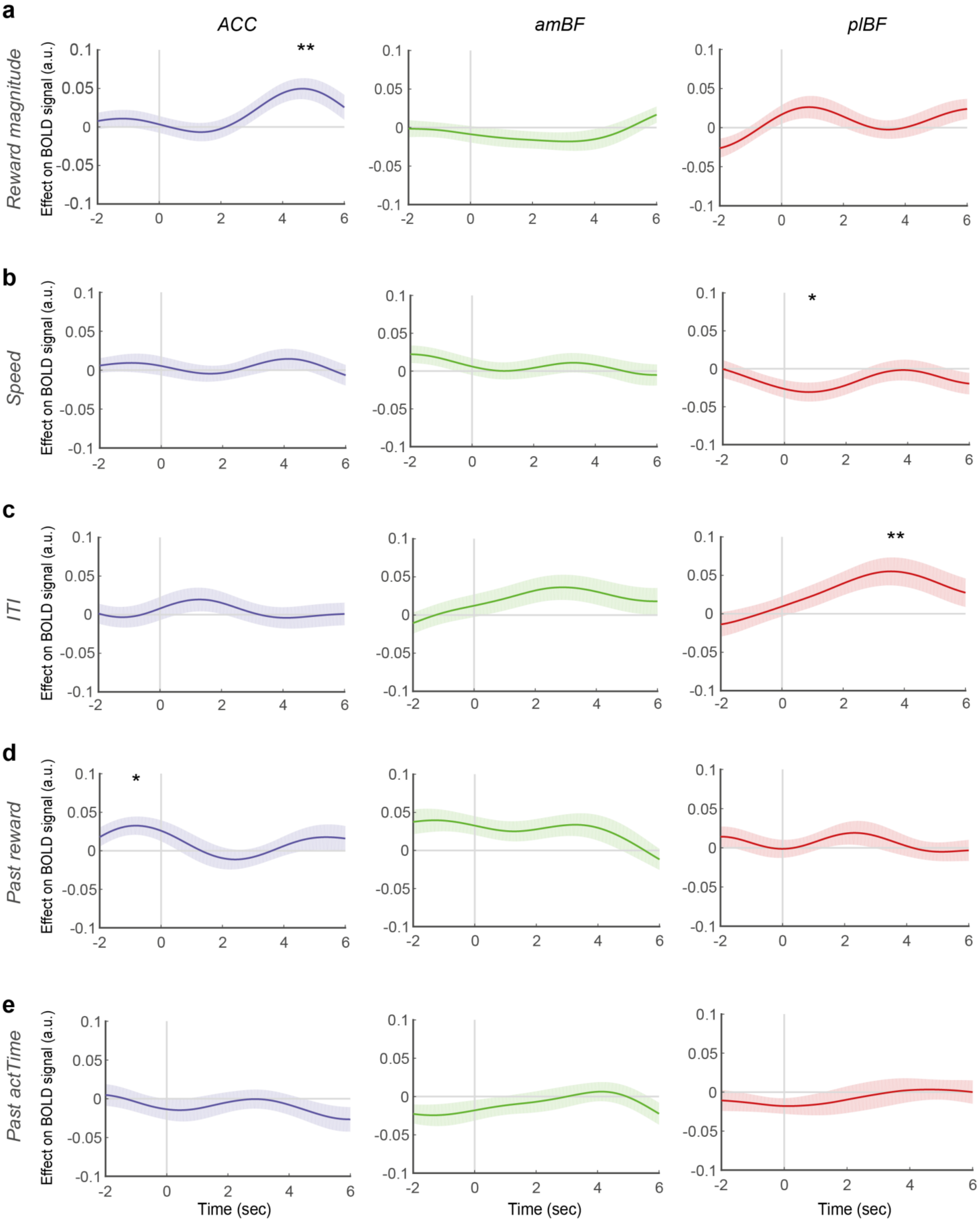

a
ACC
amBF
plBF
Reward magnitude
b
Speed
c
ITI
d
Past reward
e
Past actTime
Effect on BOLD signal (a.u.)
Time (sec)
**
*

Figure S3. Effect of contextual factors on BOLD signal. Related to Figure 4. ROI time-course analysis of the ACC (blue), amBF (green), and plBF (red), showing the relationship between BOLD activity and parametric variation in reward magnitude (a), dot speed (b), ITI (c), past reward (d), and past *actTime* (e). We found individual effect of contextual factors on ACC (reward magnitude and past reward outcome) and plBF (speed and ITI). Given that both reward magnitude and *actTime* are correlated with the ACC BOLD signal, we asked whether expected value (EV) (the product of reward magnitude and reward probability at the time of response) might be a better predictor of activity in ACC. We performed the same analysis as in GLM2.1 (see methods), however, this time we replaced *actTime* with EV. We found no significant relationship between EV and BOLD signal in ACC ($t(44)=1.19$, $P=0.24$; leave-one-out procedure for peak selection). Importantly, the effect of *actTime* on ACC BOLD signal was stronger than the effect of EV ($t(44)=2.38$, $P=0.02$; paired-samples t-test). ACC activity reflects both reward probability and magnitude and other factors determining EV in choice selection tasks (Cavanagh et al., 2016; Hunt et al., 2015; Kennerley et al., 2009). However, in the current context, in which expected reward magnitude was explicitly cued and action timing was all important and directly under the monkey's control, ACC tracked expected reward magnitude and reflected the *actTime* observed on each trial. *actTime* in turn, albeit non-linearly (via a sigmoid function), determined likelihood of reward. Importantly, a significant effect of individual contextual factors on an ROI signal does not imply that that specific ROI influences the *relationship* between the contextual factor and *actTime*. Instead it simply indicates a direct effect of a contextual factor on ROI BOLD signal, regardless of its influence on *actTime*. We discuss the relationship between contextual factors and *actTime,* and their influence on BOLD signal, later in Figures 5 and 6. The lines and shadings show the mean and standard error of the β weights across the sessions, respectively. Time zero is the response time. Significance testing on time-course data was performed by using a leave-one-out procedure on the group peak signal. One-sample t-tests, * $P<0.05$, ** $P<0.01$, *** $P<0.001$.

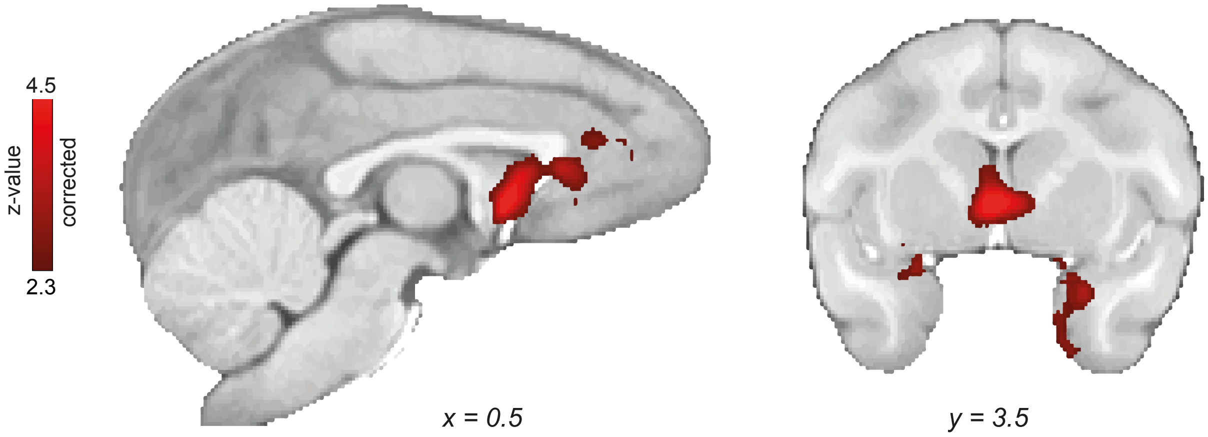

Figure S4. Whole-brain analysis showing voxels where activity reflected parametric variation in the deterministic *actTime*. Related to Figure 5. To verify whether areas outside our ROIs could also encode deterministic *actTime*, we performed a model-based whole-brain analysis. This confirmed that voxels that are correlated with trial-by-trial variation in deterministic *actTime* mainly overlap with the BF ROI. Format as in Fig.S2a, but after replacing observed *actTime* with deterministic *actTime* as predicted by Cox model. Two significant clusters were identified: the peak of the largest cluster was at BF (peak Z=5.1, number of voxels=9203; F99: x=1.0, y=4.0, z=2.0; whole-brain cluster-based correction, Z>2.3, P<0.001). The smaller cluster was at the boundary of the left amygdala and entorhinal cortex (peak Z=5.1, number of voxels=2197; F99: x=-9.0, y=4.5, z=-7.0; whole-brain cluster-based correction, Z>2.3, P=0.003).

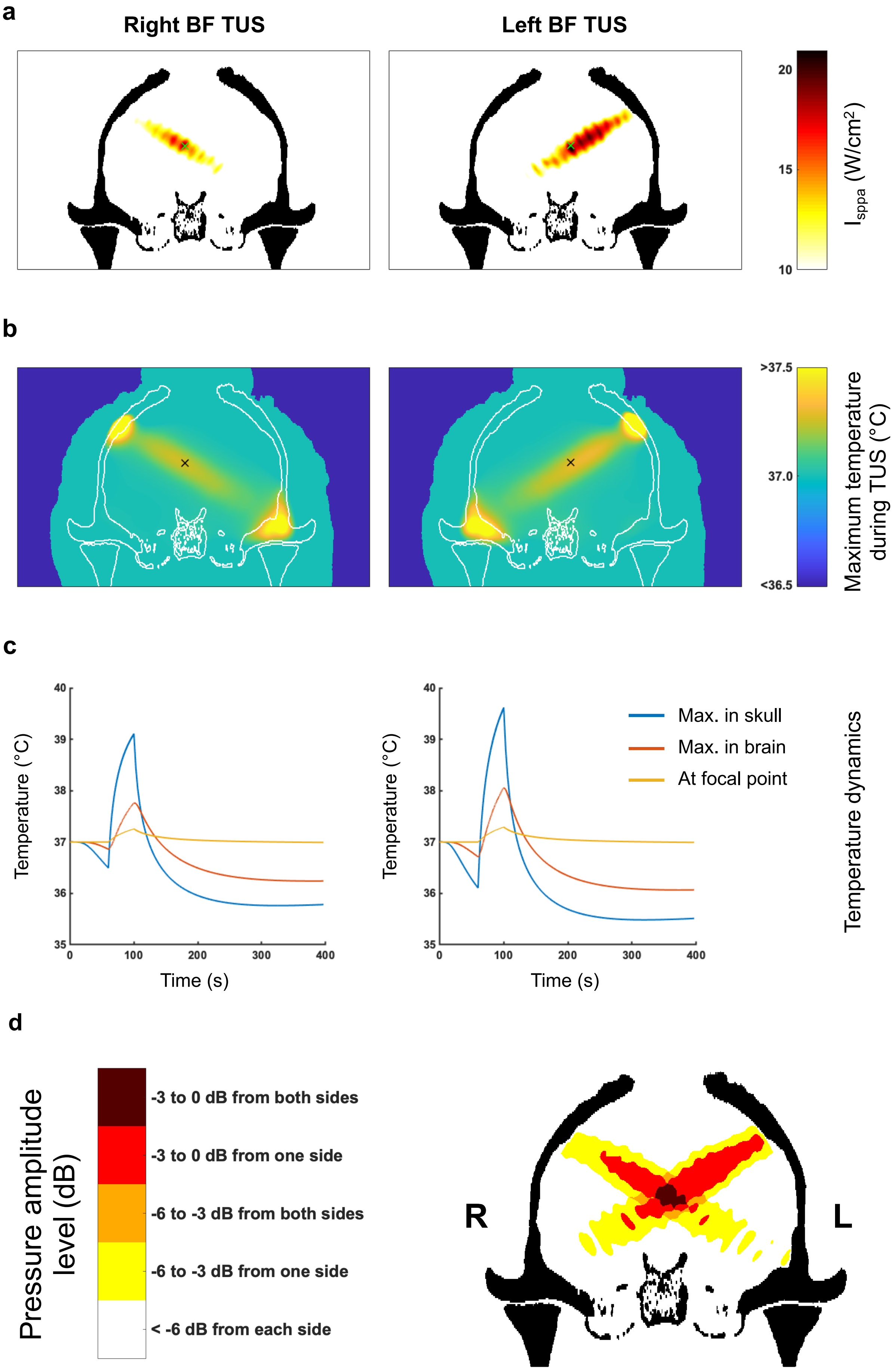

a
Right BF TUS
Left BF TUS
20
15
10
I_{sppa} (W/cm²)
b
>37.5
37.0
<36.5
Maximum temperature during TUS (°C)
c
Temperature (°C)
Time (s)
Max. in skull
Max. in brain
At focal point
Temperature dynamics
d
Pressure amplitude level (dB)
-3 to 0 dB from both sides
-3 to 0 dB from one side
-6 to -3 dB from both sides
-6 to -3 dB from one side
< -6 dB from each side
R
L

Figure S5. Simulation of the acoustic wave propagation and its thermal effect. Related to Figure 7. (a-c) Peak intensities, spatial distribution and thermal modelling for right BF targeting (left column) and left BF targeting (right column). (a) Simulated focused ultrasound peak intensities and spatial distribution in the brain when targeting BF, based on a high-resolution macaque whole-head CT scan. The maximum spatial-peak pulse-averaged intensity (I_{sppa}) at the acoustic focus point was 21.2 W/cm^2 for the left BF target and 18.9 W/cm^2 for the right BF target (spatial peak temporal average intensities, I_{spta}: 6.4 W/cm^2 and 5.6 W/cm^2 for left and right, respectively). (b) Whole-head modelling of the maximum thermal rise during 40 s TUS. BF stimulation target position is shown as a cross for both sides on sagittal views ((a) and (b)). (c) Temperature dynamics for the hottest point in the skull (blue), the hottest point in the brain (red) and the geometrical focal point in the brain (yellow). Given that the skull is more acoustically absorbing than soft tissue, the highest thermal increase is located in the skull itself, estimated by the simulation to be 2.6°C and 2.1°C for left and right, respectively. For an approximate 0.5 mm thickness of the dura the maximum temperature in the brain below the dura was 38.1°C and 37.8°C for left and right, respectively. The maximal thermal increase at the geometrical focus of the sonic transducer was less than 0.3 °C for both sides. (d) Representation of pressure amplitude overlap from successive left and right BF TUS. Pressure fields were obtained from whole-head simulations of the focused ultrasound pressure amplitude when targeting left and right BF, based on a high-resolution macaque whole-head CT scan. The pressure fields are superimposed and are represented in relative pressure levels where 0 dB is the maximum pressure amplitude estimated in the brain. Dark red and orange highlight areas where the pressure is higher than -3dB and -6dB, respectively, for both sonications due to an overlap of the two beams. Red and yellow highlight areas where the pressure is higher than -3dB and -6dB, respectively, for one of the sonications.

a

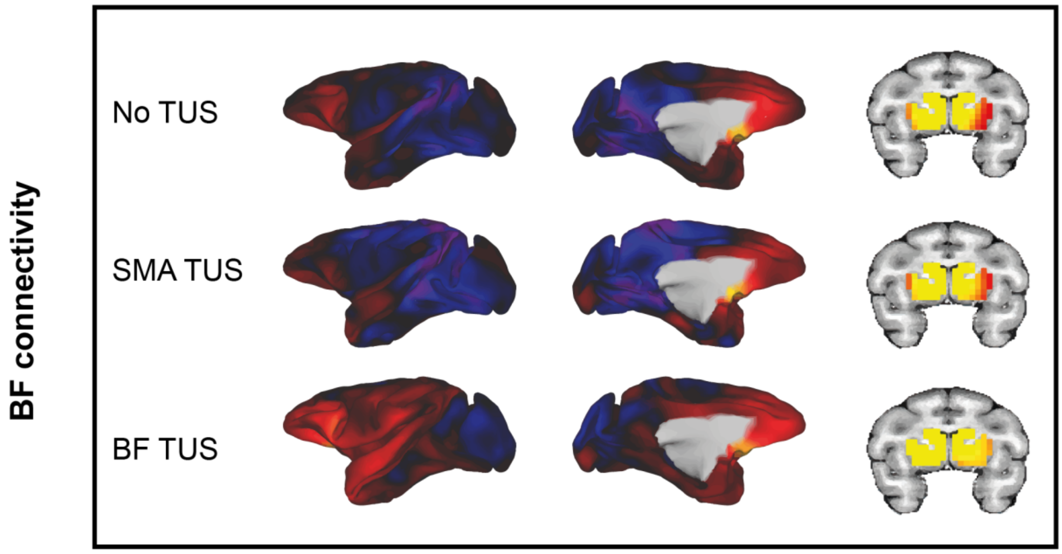
BF connectivity
No TUS
SMA TUS
BF TUS

b

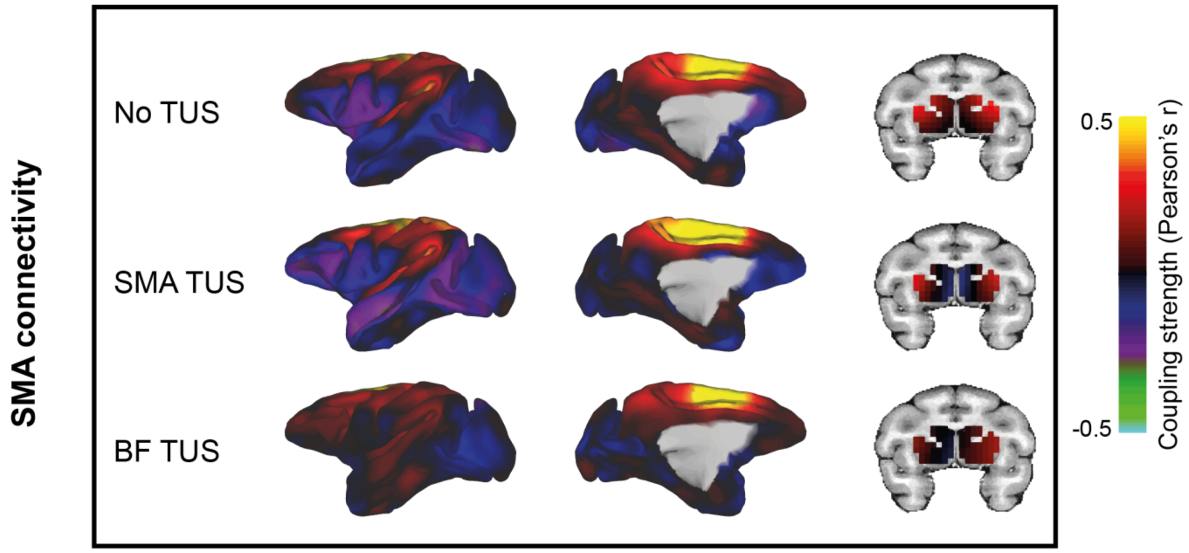
SMA connectivity
No TUS
SMA TUS
BF TUS
0.5
-0.5
Coupling strength (Pearson's r)

c

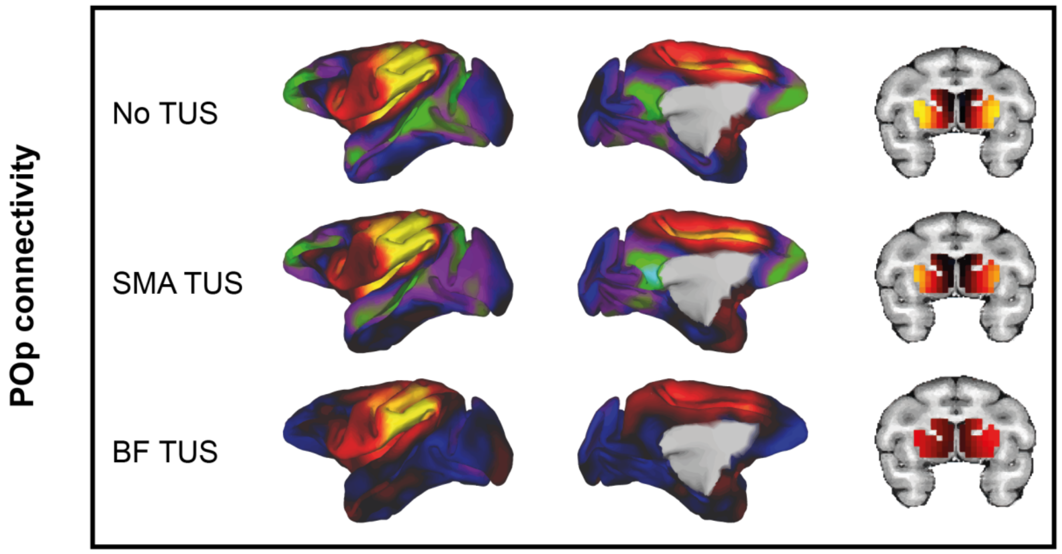
POp connectivity
No TUS
SMA TUS
BF TUS

Figure S6. Effects of TUS were specific to stimulation of BF. Related to Figure 7.

Whole-brain functional connectivity of the BF (a), a control region in the SMA (b), and a control region in POp (c). In each panel the upper row shows functional connectivity from the seed area to the rest of the brain without applying TUS. The middle and bottom rows show seed-based connectivity after SMA and BF TUS, respectively. Positive correlations are represented in warm colours from red to yellow, negative correlations are represented in cool colours from blue to green. After TUS over BF, BF positive coupling (a) is enhanced within BF and between BF and frontal and cingulate gyri. Importantly, this pattern is not observed when looking at the connectivity between SMA (b) or POp (c) and the rest of the brain. In fact, panels b and c show a widespread suppression of coupling elsewhere in the brain after BF TUS, compared to no TUS and SMA TUS.

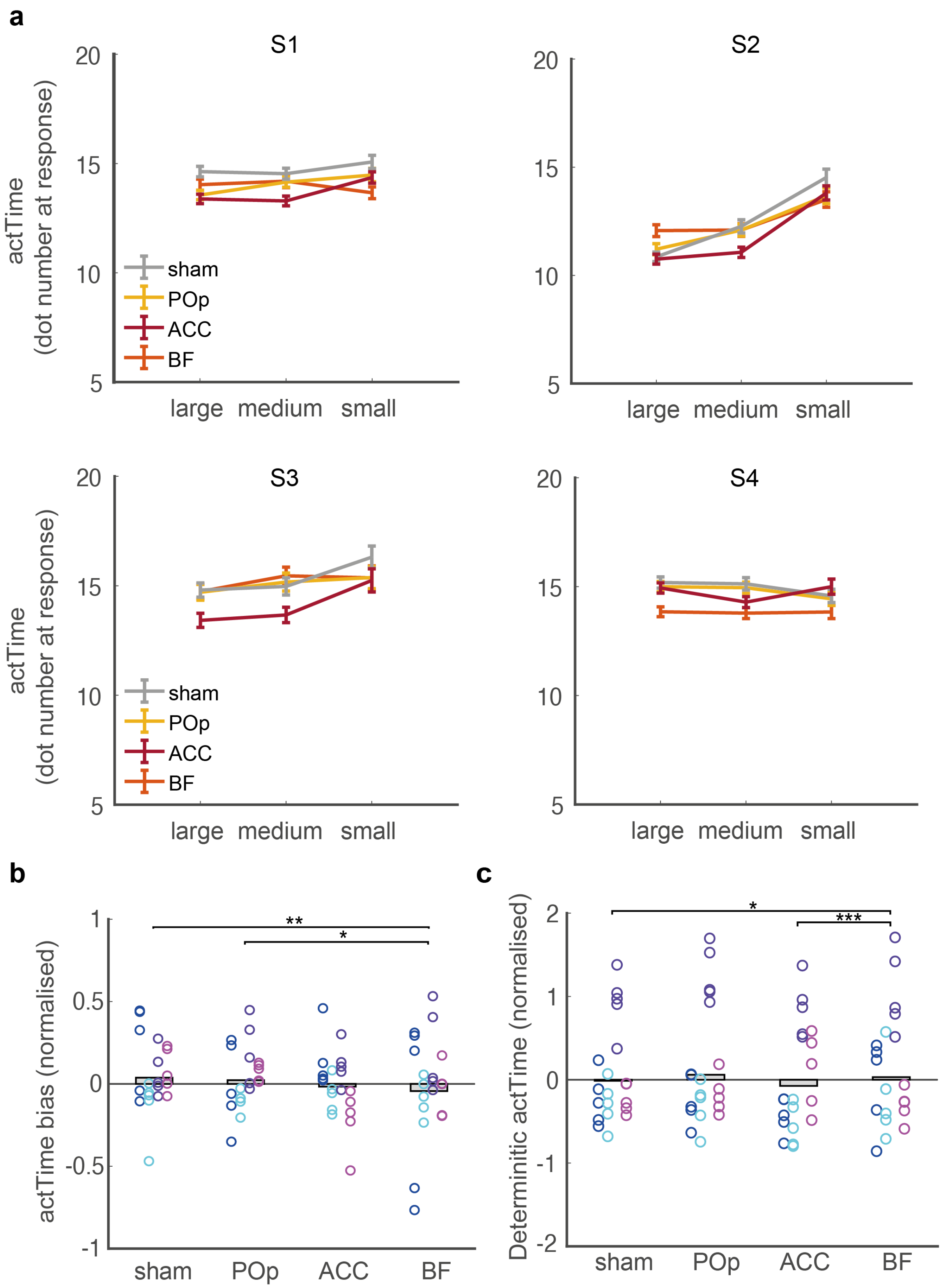
a
S1
S2
S3
S4
actTime
(dot number at response)
20
15
10
5
sham
POp
ACC
BF
large medium small
b
actTime bias (normalised)
1
0.5
0
-0.5
-1
**
*
sham POp ACC BF
c
Determinitic actTime (normalised)
2
1
0
-1
-2
*
***
sham POp ACC BF

Figure S7. Behavioural effects of TUS. Related to Figure 7. (a) Animals acted quicker after ACC than after BF, POp or sham TUS, when offered medium or large, compared to small rewards. Format as in Fig.7di but with data from each animal (S1, S2, S3, S4) reported separately. Error bars show standard error of the mean. (b-c) The effect of BF TUS on *actTime bias* (b) and deterministic *actTime* (c). *actTime bias* was defined as the trial-by-trial absolute difference between observed and deterministic *actTime*. We found a statistically significant effect of TUS on *actTime bias,* however, careful inspection of the data shows that the effect is not consistent across all four animals. We could not include by-subject random slope in the model as it failed to converge due to our small sample size (n=4). Further analyses of each individual animal's data revealed that in two animals there was a significant main effect of TUS location on *actTime bias* (S1, $X^2(3)=15.94$, $P=0.001$; S4, $X^2(3)=14.86$, $P=0.002$) and in one animal there was a significant interaction between the effect of TUS location and time (the effect of BF TUS became more marked as the session progressed) (S2, $X^2(3)=10.54$, $P=0.015$). The effect was not significant in the fourth animal (S3). Format as in Fig.7e but with data from each animal and each stimulation session reported separately. Each colour represents one animal, and each ring is one stimulation session. The black grey columns illustrate the group mean across all observations. Multilevel ANOVA followed by pairwise t-test. * $P<0.05$, ** $P<0.01$, *** $P<0.001$.

Cluster	P	Z-max	Z-max X (mm)	Z-max Y (mm)	Z-max Z (mm)	COPE-mean
BF	0.008	4.5	4.5	2.0	-2.0	54.1
ACC	0.004	3.9	0.5	20.5	12.5	54.8
Right anterior insula	<0.001	3.9	19.6	3.0	-5.0	49.7
Left putamen	< 0.001	3.9	-10.5	-1.5	5.0	45.1

Table S1. List of all clusters. Related to Figure 4. We found four significant clusters at threshold Z > 2.3. In this study we focused on BF and ACC with bilateral/midline activity.
